# Parallel genome-wide screens identify synthetic viable interactions between the BLM helicase complex and Fanconi anemia

**DOI:** 10.1038/s41467-017-01439-x

**Published:** 2017-11-01

**Authors:** Martin Moder, Georgia Velimezi, Michel Owusu, Abdelghani Mazouzi, Marc Wiedner, Joana Ferreira da Silva, Lydia Robinson-Garcia, Fiorella Schischlik, Rastislav Slavkovsky, Robert Kralovics, Michael Schuster, Christoph Bock, Trey Ideker, Stephen P. Jackson, Jörg Menche, Joanna I. Loizou

**Affiliations:** 1 CeMM Research Center for Molecular Medicine of the Austrian Academy of Sciences, Lazarettgasse 14, AKH BT 25.3, 1090 Vienna, Austria; 2 Department of Medicine, Division of Genetics, University of California San Diego, La Jolla, CA 92093 USA; 3 Department of Bioengineering, University of California San Diego, La Jolla, CA 92093 USA; 4 Moores Cancer Center, University of California San Diego, La Jolla, CA 92093 USA; 5 The Cancer Cell Map Initiative, La Jolla, CA 92093 USA; 6 The Wellcome Trust and Cancer Research UK Gurdon Institute, and Department of Biochemistry, University of Cambridge, Cambridge, CB2 1QN UK; 7 The Wellcome Trust Sanger Institute, Hinxton, Cambridge, CB10 1SA UK; 8 Present Address: Institute of Molecular and Translational Medicine, Faculty of Medicine and Dentistry, Palacky University Olomouc, Olomouc, Czech Republic

## Abstract

Maintenance of genome integrity via repair of DNA damage is a key biological process required to suppress diseases, including Fanconi anemia (FA). We generated loss-of-function human haploid cells for FA complementation group C (FANCC), a gene encoding a component of the FA core complex, and used genome-wide CRISPR libraries as well as insertional mutagenesis to identify synthetic viable (genetic suppressor) interactions for FA. Here we show that loss of the BLM helicase complex suppresses FANCC phenotypes and we confirm this interaction in cells deficient for FA complementation group I and D2 (FANCI and FANCD2) that function as part of the FA I-D2 complex, indicating that this interaction is not limited to the FA core complex, hence demonstrating that systematic genome-wide screening approaches can be used to reveal genetic viable interactions for DNA repair defects.

## Abstract (web summary)

Fanconi anemia is a complex disease affecting multiple DNA repair proteins that resolve DNA crosslinks which can block vital processes. Here the authors use parallel genome-wide screens that identify the BLM helicase complex as a suppressor of Fanconi anemia phenotypes.

## Introduction

Maintaining genome integrity via repair of DNA damage is a key biological process required to suppress diseases including cancer, ageing-related pathologies and diseases associated with developmental defects and neurological disorders^1,2^. Defects in DNA repair genes cause various rare heritable diseases. One such disease is Fanconi anemia (FA) that is caused by defects in FA genes and is characterized by bone marrow failure, congenital defects, cancer predisposition and chromosome fragility^3^. FA is believed to result from impaired repair of DNA interstrand crosslink (ICL) damage, leading to accumulation of DNA damage and genome instability. Furthermore, FA patients that develop cancer cannot be treated with standard chemotherapy, including crosslinking agents, as they are hypersensitive to such compounds.

Synthetic viability is the suppression of a genetic defect or phenotype by mutation or abrogation of another gene or pathway. Recently, haploid genetic screens have emerged as a powerful method to perform suppression screens in human cells^4–6^. Using near-haploid cell lines, such as HAP1, in combination with a CRISPR-Cas9 inactivating library and insertional mutagenesis, knock-outs for nearly all non-essential human genes can be generated^7,8^.

Here, we introduce an approach for the systematic identification of synthetic viable interactions in human cells, illustrated with FA defective cells. We identified synthetic viable interactions for FA by performing genome-wide screens on isogenic human haploid cells lacking the FA complementation group C (FANCC) protein, following exposure to the DNA ICL-inducing agent mitomycin C (MMC). We identify the BLM helicase complex as a suppressor of Fanconi anemia phenotypes in human cells, demonstrating that systematic screening approaches can be used to reveal genetic viable interactions for DNA repair defects.

## Results

### Genome-wide screens identify synthetic viable interactions

To validate the use of HAP1 as a cellular model system in which to identify genetic synthetic viable interactions for genes associated with DNA repair, we reproduced a reported synthetic viable interaction that occurs between lamin A (mutated in the premature-ageing disease Hutchinson-Gilford progeria syndrome) and the acetyl-transferase protein NAT10^9^. Hence, we utilized CRISPR-Cas9 lamin A mutant HAP1 cells (Δ*LMNA*) (Supplementary Fig. 1a) which displayed a misshaped nuclear morphology that could be corrected upon the addition of a NAT10 inhibitor (Remodelin) (Supplementary Fig. 1b). Next, we targeted *FANCC* in HAP1 cells using CRISPR-Cas9, generating a frame-shift mutation (Supplementary Fig. 1c) and subsequently the loss of FANCC protein expression (Supplementary Fig. 1d). Resulting *FANCC* mutant cells (Δ*FANCC*) were hypersensitive to MMC, both in a short-term dose-response assay (Supplementary Fig. 1e) and in a long-term colony formation assay (Supplementary Fig. 1f, g).

**Fig. 1:**
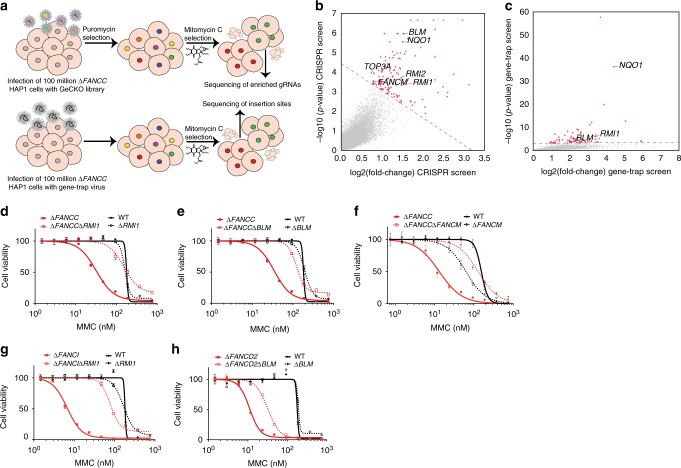
Genome-wide CRISPR-Cas9 and insertional mutagenesis screens identify the BLM complex as a synthetic viable interaction for FANCC. **a** Workflow for the identification of genetic synthetic viable interactions for *∆FANCC* cells following MMC exposure by two parallel genome-wide approaches: CRISPR-Cas9 and insertional mutagenesis. **b** Viability-inducing genes identified using a genome scale CRISPR knock-out (GeCKO) library in ∆*FANCC* cells treated with MMC, compared to untreated WT cells are shown in red, and include members of the BLM complex, *FANCM* and *NQO1*. Each dot represents the average score of the six guide RNAs (gRNAs) per gene. **c** Viability-inducing genes identified using gene-trap insertional mutagenesis in ∆*FANCC* cells treated with MMC, compared to untreated WT cells. Members of the BLM complex and *NQO1* are labeled. For robust identification of enriched genes in **b**, **c**, hit selection was performed in two steps. First, each data set was partitioned into two groups, defining the hit-group as data points with *p* < 0.001 and fold-change >2^1.5^. In a second step, hit selection was optimized using linear discriminant function analysis. **d**–**h** Indicated cell lines were exposed to MMC for 4 days and cellular survival was assessed by CellTiter-Glo. Means and S.E.M. of biological triplicates are plotted

To identify synthetic viable interactions for FANCC, we set up two genome-wide approaches to screen for mutations that alleviate the hypersensitivity of Δ*FANCC* cells to MMC-induced DNA damage (Fig. 1a). To this end, we exposed these cells to the Genome-Scale CRISPR Knock-Out (GeCKO) library^10^ or insertional mutagenesis^8^, the latter disrupting genes by random insertion of a gene-trap cassette into the genome. Cells were subsequently grown under MMC selection, leaving 5–10% of ∆*FANCC* cells viable. Cells resistant to MMC were recovered and subjected to next generation sequencing, to identify either the enriched guide RNAs (gRNAs) or positions of insertional gene-trap mutagenesis. Sequencing of the CRISPR library revealed a sufficient number of reads, covering each gRNA around 300 times (Supplementary Fig. 2a, b **)**. More than 99% of all gRNAs present in the CRISPR library were detected (Supplementary Fig. 2c). Use of insertional mutagenesis resulted in the targeting of >7000 genes with a total number of 22,772 unique insertions (Supplementary Table 1). For both genome-wide screens, the CRISPR-Cas9 mediated editing and insertional mutagenesis screen, we used human haploid HAP1 cells since the likelihood to receive loss-of-function mutations is increased by the fact that only one genetic allele needs to be altered to yield a null phenotype^4,5,8,11^. All experiments confirming the results of the genome-wide screens were performed using diploid HAP1 clones.

Encouragingly, both approaches led to the identification of NAD(P)H:quinone oxidoreductase1 (NQO1) as highly enriched in *ΔFANCC* cells treated with MMC, compared to untreated wild-type (WT) cells (Fig. 1b, c). NQO1 functions as a positive control, since it is known that loss of NQO1 renders cells less sensitive to MMC due to its functions as one of several bioreductases, converting MMC from a pro-drug to an active form that can lead to ICLs^12^. Moreover, *NQO1* is found to carry loss-of-function mutations in cancers that are MMC resistant^13^. Using the CRISPR library, as well as insertional mutagenesis, we identified the enrichment of several NQO1 gRNAs and multiple *NQO1* inactivating insertions, respectively (Supplementary Fig. 3a, b). To validate this genetic interaction, we designed gRNAs to target NQO1 with Cas9 nickase^14^ (Supplementary Fig. 3c) and confirmed that editing resulted in a pool of frame-shift mutations by immunoblotting (Supplementary Fig. 3d). Both WT and *ΔFANCC* cells targeted for NQO1 (‘WT + *NQO1* gRNA’ and ‘*ΔFANCC* + *NQO1* gRNA’, respectively) displayed reduced MMC toxicity in both a short-term dose-response assay (Supplementary Fig. 3e) and a long-term colony formation assay (Supplementary Fig. 3f, g).

### Loss of BLM complex rescues sensitivity of FA cells to ICLs

We identified several members of the BLM complex, using both genome-wide CRISPR libraries (where we identified all four complex members: BLM, RMI1, RMI2 and TOP3A) and insertional mutagenesis (where we identified BLM and RMI1) (Fig. 1b, c), and followed up on this finding. The BLM complex forms part of a multienzyme DNA helicase and includes DNA Topoisomerase III Alpha (TOP3A), RMI1, RMI2, and the BLM helicase. The BLM complex is bridged to the FA complex via FANCM^15^, and indeed gRNAs targeting *FANCM* were also enriched using the CRISPR library (Fig. 1b and Supplementary Fig. 4a). The BLM complex functions in the resolution of DNA structures that arise during the process of homologous recombination (HR) repair^16^. By comparing enriched genes in the CRISPR screen performed on MMC-treated *ΔFANCC* cells to enriched genes identified by an additional CRISPR screen performed on MMC-treated WT cells, we found that loss of the BLM complex specifically rescued *ΔFANCC* but had little or no effect in WT cells (Supplementary Fig. 4a). This indicates that the observed phenotype of increased resistance upon loss of BLM is specific to FANCC deficient cells and most likely does not result from general pro-survival effects due to diminished MMC uptake, impaired apoptotic signaling or perturbed MMC activation. All six gRNAs for *BLM* and *RMI1* were enriched in the CRISPR screen (Supplementary Fig. 4b). In addition, inactivating insertion sites within *BLM* and *RMI1* in the gene-trap screen were identified (Supplementary Fig. 4c).

To validate the above findings, we generated BLM, RMI1 or FANCM deficient cells both in a WT background and in a *ΔFANCC* background. Single and double knock-out clones were confirmed by Sanger sequencing (Supplementary Fig. 4d) and immunoblotting (Supplementary Fig. 4e). Thus we confirmed that while loss of RMI1, BLM or FANCM in a WT background (*ΔRMI1*, *ΔBLM*, and *ΔFANCM*, respectively) had little effect on MMC sensitivity, loss of one of these three factors in a FANCC-deficient background (*ΔFANCCΔBLM*, *ΔFANCCΔRMI1*, and *ΔFANCCΔFANCM*) resulted in enhanced MMC resistance (Fig. 1d–f). In support of our findings, it has been reported that disruption of *FANCM* (Protein Hef ortholog) in a FANCC-deficient background in chicken DT40 cells suppresses cellular sensitivity to cisplatin^17^. In addition, it has been shown that mouse embryonic fibroblasts lacking both FANCB (another member of the FA core complex) and BLM are less sensitive to MMC than FANCB single mutants^18^. However, intriguingly chicken DT40 cells lacking both FANCC and BLM are not noticeably less sensitive than FANCC single mutants^19^ and this discrepancy may be due to species variation.

Since FANCC is part of the FA core complex, we next investigated whether loss of BLM or RMI1 could rescue cells lacking FANCI and FANCD2, that make up the FA I-D2 complex and function downstream of the FA core complex. To this end, we generated FANCI and FANCD2 mutant HAP1 cells (*ΔFANCI and ΔFANCD2*), as well as ∆*FANCI*∆*RMI1* and ∆*FANCD2*∆*BLM* double-deficient cells (Supplementary Fig. 4f, g). We observed that loss of RMI1 or BLM could rescue the MMC hypersensitivity of FANCI and FANCD2 deficient cells, but did not enhance MMC resistance in WT cells (Fig. 1g, h), indicating that this synthetic viable (genetic suppression) interaction is not limited to FA core complex components.

**Fig. 2:**
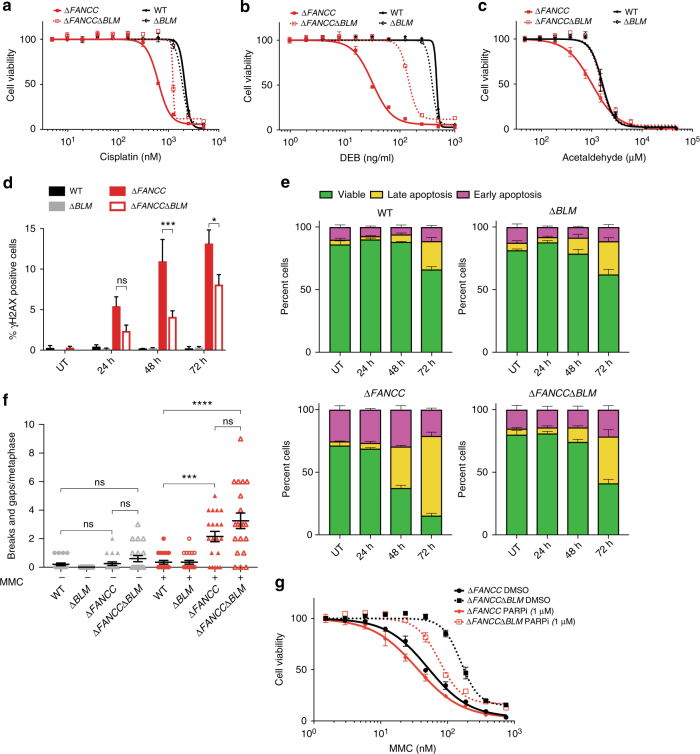
Loss of the BLM complex in FANCC deficient cells alleviates DNA damage and apoptosis induced by ICLs. **a**–**c** Treatment of indicated cell lines with cisplatin, diepoxybutane (DEB) and acetaldehyde for 4 days. Survival assessed by CellTiter-Glo. **d** Indicated cells were left untreated (UT) or treated with MMC for 24, 48, or 72 h, stained for γH2AX and analyzed by High Content Imaging. *p*-values determined by two-way ANOVA. **e** Cells were either left untreated or treated with MMC for 24, 48, and 72 h, then apoptosis was measurement using propidium iodide (PE) Annexin V staining, followed by flow cytometry analysis. **f** Quantification of chromosome breaks and gaps of 40 cells per cell line treated with MMC for 24 h followed by analysis of metaphase spreads. *p*-value determined by Mann–Whitney U test. **g** Survival of indicated cells treated with either the PARP inhibitor olaparib (PARPi) or DMSO for 4 h, followed by MMC exposure for 4 days. Survival assessed by CellTiter-Glo. For all panels, means and S.E.M. of biological triplicates are plotted. ns = *p* > 0.05; ^*^=*p* < 0.05; ^**^=*p* < 0.01; ^***^=*p* < 0.001; ^****^=*p* < 0.0001

To investigate whether the observed genetic interaction between FA and the BLM complex was specific to MMC, we treated cells with two additional crosslinking agents, cisplatin and 1,2,3,4-diepoxybutane (DEB) (Fig. 2a, b). In addition, we treated cells with acetaldehyde, which is considered to be an endogenous source of crosslinking damage in FA cells^20–22^ (Fig. 2c). We noted that loss of BLM alleviated the cellular hypersensitivity of *∆FANCC* cells to all of these crosslinking agents.

### p53 is partially functional in HAP1 cells

Because DNA-damage induced cell death of FA cells has been shown to occur in a p53 dependent manner^23^, and since we did not retrieve *TP53* or its effectors in either of the genome-wide loss-of-function screens, we next investigated the functionality of p53 in HAP1 cells. Sequencing of *TP53* confirmed a previously reported point mutation^24,25^ (Supplementary Fig. 5a). To test whether this mutation impacted on cell survival of FA cells following exposure to MMC, we generated HAP1 cells lacking p53 (∆*TP53)* as well as cells lacking both FANCC and p53 *(∆FANCC∆TP53)* (Supplementary Fig. 5b, c). Next, we treated these cells with MMC in both short-term and long-term dose-response assays (Supplementary Fig. 5d–f). We noted that loss of p53 only slightly increased the resistance of *∆FANCC* cells to MMC **(**Supplementary Fig. 5d–f **)**. This led us to address p53 functionality in HAP1 cells. Thus, we treated human A549 cells (a *TP53* WT cell line) and HAP1 cells with Nutlin-3a, an MDM2 inhibitor. This enhanced p53 stability in A549 cells and ensuing induction of the p21 protein (Supplementary Fig. 5g). While we did observe low levels of p21 protein in HAP1 cells, this was not increased upon Nutlin-3a treatment, nor did Nutlin-3a increase p53 protein levels in such cells. Next, we tested whether Nutlin-3a treatment could sensitize HAP1 cells in both short-term and long-term dose-response assays (Supplementary Fig. 5h–j). We did not observe an increased sensitization to Nutlin-3a, which taken together with our other data, these findings indicate that p53 is only partially functional in HAP1 cells.

### Loss of BLM reduces DNA damage and apoptosis of FA cells

To probe the molecular mechanism of the observed suppression, we measured generation and clearance of DNA damage using Ser-139 phosphorylated histone H2AX (γH2AX) as a marker (Fig. 2d). This analysis indicated a reduction in DNA damage at later time points (48 and 72 h) in *∆FANCC∆BLM* cells compared to *∆FANCC* cells which correlated with reduced levels of apoptosis (Fig. 2e and Supplementary Table 2). Next, we measured the impact of loss of BLM in *∆FANCC* cells on chromosomal instability by assessing chromosomal aberrations (Fig. 2f). This indicated that shortly after ICL induction (24 h after MMC treatment), the number of chromosomal breaks and gaps in *∆FANCC∆BLM* cells were not significantly altered, compared to *∆FANCC*.

### Loss of BLM does not rescue general HR defects

DNA double-strand breaks are generated as an intermediate structure during ICL repair and are repaired through HR^26^. To test whether loss of BLM can rescue the DNA-damage hypersensitivity of cells defective in other HR proteins, we tested whether effects resulting from depletion of BRCA1 could be compensated for by loss of BLM. Hence, we depleted BRCA1 in WT and *∆FANCC* cells, then exposed them to MMC (Supplementary Fig. 6a–c). These data indicated that loss of BLM did not alleviate the sensitivity of BRCA1 depleted cells to MMC, indicating that this genetic interaction with BLM is specific to the FA pathway.

### MUS81 loss does not sensitize ∆*FANCC*∆*BLM* cells to MMC

The BLM complex, also known as ‘dissolvasome’, can dissolve catenated DNA structures that arise during replication and HR repair^27^. The helicase activity of BLM in combination with the topoisomerase activity of TOP3A are required to dissolve the double Holliday junction that is formed during HR in a way that results in a non-crossover DNA product and prevents sister chromatid exchanges. Alternatively, the same structure can be resolved by structure-specific nucleases such as MUS81-EME1 or GEN1 that cut the DNA leading to crossover DNA products^28^. A possible hypothesis for the observed suppression of FA DNA-damage sensitivity by loss of BLM components is that in the absence of a functional FA pathway, inappropriate or incomplete processing of ICLs might result in a structure that cannot be efficiently dissolved by the helicase activity of BLM, resulting in a toxic intermediate. In the absence of the BLM complex, however, preferential resolution by structure-specific nucleases, such as MUS81-EME1, might be more efficient and promote survival. A recent study showed that BLM might contribute to the generation of chromosome breaks and radials in a FANCB deficient background, supporting this hypothesis^29^. A corollary of the above model is that loss of MUS81-EME1 function would re-sensitize *∆FANCC∆BLM* cells to DNA-damaging agents. However, when we tested this idea by depleting MUS81 using two independent shRNAs in *∆FANCC∆BLM* cells (*∆FANCC∆BLM shMUS81#1 and ∆FANCC∆BLM shMUS81#2)* (Supplementary Fig. 6d), we found that *∆FANCC∆BLM* cells depleted for MUS81 were not re-sensitized to MMC (Supplementary Fig. 6e). Thus we conclude that MUS81 appears not to be used as an alternative nuclease to BLM in the context of our analyses.

### PARP inhibition re-sensitizes *∆FANCC∆BLM* cells to MMC

Recently, a role for alternative end joining in the FA pathway has been reported^30,31^. As BLM may function in regulating the pathway choice for DNA double-strand break repair, by preventing alternative end-joining^32^, we asked whether its absence could allow for the use of this pathway in the removal of ICLs in FA-deficient cells. As alternative end-joining is known to depend on PARP, we inhibited PARP with a small molecule inhibitor olaparib, and tested cellular survival to MMC. This led to a partial sensitization of *∆FANCC∆BLM* cells, thereby suggesting that alternative end-joining may enhance survival of FA cells in the absence of BLM function by repairing some DNA lesions (Fig. 2g and Supplementary Fig. 6f).

## Discussion

Although the exact mechanisms by which the BLM complex impacts on the FA pathway still needs to be resolved, several levels of crosstalk have been described. Apart from its role in the resolution of HR intermediates, the BLM complex also plays an early role in replication fork protection and remodeling during ICL repair, since it is recruited to the site of the lesion upon recognition by FANCM^15,33^. BLM recruitment and helicase activity is important for proper downstream activation of the FA pathway^15^, while FANCD2 is required for the maintenance of BLM protein stability, for mediating phosphorylation of the BLM complex members in response to DNA damage and to cooperate with BLM to promote restart of stalled replication forks while suppressing firing of new replication origins^34^. It is thus evident that coordinated action of BLM and FA proteins is necessary for efficient processing and repair of ICLs. Here we report that when members of both complexes are absent, ICL lesions can be channeled through alternative repair pathways, at the cost of genome integrity.

BLM has also been shown to prevent CtIP- and Mre11-mediated alternative non-homologous end-joining^32^, a repair pathway that results in DNA sequence alterations and has also been shown to be involved in ICL repair^31^. Thus, loss of BLM might relieve suppression of alternative end-joining and promote repair of the lesions in an error-prone manner, a hypothesis supported by our data showing reduced γH2AX foci upon treatment with MMC, without decreasing chromosomal aberrations. This is supported by the partial sensitization of *∆FANCC∆BLM* cells to MMC upon inhibition of PARP.

In conclusion, through the use of parallel genome-wide screens, we have shown that synthetic viable (genetic suppression) interactions for Fanconi anemia can be systematically identified in human cells. We discovered that loss of the BLM complex rescues survival of Fanconi anemia deficient cells upon generation of DNA damage by reagents that generate ICLs.

## Methods

### Cell lines and culture conditions

HAP1 cells were grown in Iscove’s Modified Dulbecco’s Medium (IMDM) from GIBCO^®^, containing L-Glutamine and 25 mM HEPES and supplemented with 10% fetal bovine serum (FBS) and 1% Penicillin/Streptomycin (P/S). HEK293T cells for virus production were expanded in Dulbecco’s Modified Eagle Medium (DMEM) from GIBCO, supplemented with 10% FBS. A549 cells were grown in DMEM supplemented with 10% FBS and 1% P/S. All cells were grown at 37 °C in a 3% oxygen and 5% CO_2_ atmosphere. Diploid HAP1 clones used for all experiments (except the genome-wide screens) were obtained by serial dilution of mixed populations of cells (consisting of both haploid and diploid cells), followed by confirmation of the ploidy status by FACS. All cell lines used in this publication were tested negative for mycoplasma contamination using the MycoAlert™ Mycoplasma Detection Kit.

### Gene editing

Guide RNA pair design and cloning*:* For the generation of *NQO1*-CRISPR knock-out cells, the Cas9 double-nickase system was used^14^. By combining the CRISPR Design Tool (http://crispr.mit.edu/) and the Desktop Genetics tool (https://www.deskgen.com/landing), we selected a pair of two guide RNA (gRNAs) sequences of 20 base pairs each, targeting exon 3 of the human *NQO1* gene (ENSG00000181019) with an offset distance of 9 base pairs. The gRNA sequences used were the following: *NQO1*-guideA (Sense): 5′-TAAGCCAGAACAGACTCGGC-3′ and *NQO1*-guideB (Antisense): 5′-CCATCTGAGCCCAGATATTG-3′. The gRNA oligonucleotides were annealed and cloned into the pSpCas9n(BB)-2A-Puro (PX462) V2.0 vector (Addgene plasmid # 62987), following the recommended protocol^35^.

Plasmid transfection: pSpCas9n(BB)-2A-Puro-NQO1-guideA and -guideB constructs were co-transfected into HAP1 cells using Xfect transfection reagent (Takara Bio USA, Inc.). After 2 days of selection with puromycin, loss of protein expression was tested by immunoblotting.

CRISPR-Cas9-mediated editing: *ΔLMNA* and HAP1 cells were purchased from Horizon Genomics. CRISPR-Cas9 knock-outs of FANCC, FANCI and BLM were generated in collaboration with Horizon Genomics. CRISPR-Cas9 knock-outs of RMI1, FANCM, FANCC/BLM, FANCC/RMI1, FANCI/RMI1, and FANCD2/BLM were generated using the protocol of Horizon Genomics. Sequences for gRNAs were designed by Horizon Genomics or with the use of http://crispr.mit.edu/ and https://www.deskgen.com/landing/, respectively. Sequences of gRNAs used were:

FANCC: 5′-GCCAACAGTTGACCAATTGT-3′;

FANCI: 5′-GTATCCAGTTGGTGGAATCG-3′;

FANCM(1): 5′-AAAGACCTTTATTGCCGCCG-3′;

FANCM(2): 5′-GGTCTACACAAGCTTCCACC-3′;

BLM: 5′-AGATTTCTTGCAGACTCCGA-3′;

RMI1: 5′-ATGTTAAAGTACCTCCGATG-3′;

P53: 5′-TCCTCAGCATCTTATCCGAG-3′;

### Sanger sequencing

Genomic DNA was extracted using the Viagen Biotech DirectPCR Lysis Reagent (Cell) according to the manufacture’s protocol. Genomic regions around the gRNA-targeted sequences were amplified using the following primer pairs:

*FANCC*-For: 5′-CAAACCTACACACACATACATGGAC-3′;

*FANCC*-Rev: 5′-ACTAAACAAGAAGCATTCACGTTCC-3′;

*FANCI*-For: 5′-CTTTTTCAAAGCCCTTAACCATTGC-3′;

*FANCI*-Rev: 5′-CCCTCAACAAATTACAAACCCTCAA-3′;

*FANCM*(1)-For: 5′-CGGACGATGATGTGTTGCTT-3′;

*FANCM*(1)-Rev: 5′-CGATCTGCTGTGTCACCAAG-3′;

*FANCM*(2)-For: 5′-AGTCCTAGATAAGTGCCAGCT-3′;

*FANCM*(2)-Rev: 5′-TATTTCAGCAGCGGGACAAG-3′;

*BLM*-For: 5′-GAGCAGTGCTTACTCTTACAAAGTG-3′;

*BLM*-Rev: 5′-GTTACCGAAGACTTTTCCTTCAGTG-3′;

*RMI1*-For: 5′-AAAAATCTAAAGGGTGTGCCTGTC-3′;

*RMI1*-Rev: 5′-TGCCATCGGGTAAAAGAGGATG-3′;

*P53*-For: 5′-TTATAGGGAGGTCAAATAAGCAGCA-3′;

*P53*-Rev: 5′-ATCTACAAGCAGTCACAGCACAT-3′;

The following sequencing primers were used:

*FANCC*: 5′-ACTAAACAAGAAGCATTCACGTTCC-3′;

*FANCI:* 5′-CTTTTTCAAAGCCCTTAACCATTGC-3′;

*FANCM*(1): 5′-CGATCTGCTGTGTCACCAAG-3′;

*FANCM*(2): 5′-TATTTCAGCAGCGGGACAAG-3′;

*BLM* 5′-GTTACCGAAGACTTTTCCTTCAGTG-3′;

*RMI1* 5′-TGCCATCGGGTAAAAGAGGATG-3′;

*P53*: 5′-TTATAGGGAGGTCAAATAAGCAGCA-3′;

PCR amplification conditions: heat lid 110 °C; 94 °C 2 min; loop 35× (94 °C 30 s; 55 °C 30 s; 68 °C 1 min) 68 °C 7 min. Frameshift mutations were identified using Nucleotide BLAST against the reference genome GCF_000001405.33.

### BRCA1 and MUS81 knock-down by shRNA

*BRCA1* knock-down: The shRNA constructs for *BRCA1* were kindly provided by Sebastian M. Nijman (Ludwig Institute for Cancer Research Ltd, UK). HAP1 cells were infected with the virus-containing supernatant in the presence of polybrene (final concentration 8 μg/ml), in IMDM (10% FBS, 1% P/S), with a viral supernatant to medium ratio of 1:3. Infected cells were selected using puromycin (2 μg/ml; Sigma-Aldrich) for 48 h. *MUS81* knock-down: Targeting sequences for MUS81 were selected using the Broad Institute Genetic Perturbation Platform. Sense targeting sequences: shMUS81#1: 5′-ACACTGCTGAGCACCATTAAG-3′; shMUS81#2: 5′-CACGCGCTTCGTATTTCAGAA-3′. Oligos were cloned into the “pLKO.2 stuffer” vector, provided by Sebastian M. Nijman. Virus was produced using the pCMV-VSV-G envelope plasmid (Addgene # 8454) and the psPAX2 packaging plasmid (Addene # 12260) in HEK293 cells. For infection, 150 ul viral supernatant was added to HAP1 cells 1 ml IMDM, following the Lipofectamine® 2000 Transfection protocol.

### Quantitative reverse transcription PCR (RT-PCR)

Cells were collected and RNA was isolated using Trizol extraction (following manufactures instructions). RNA was treated with 1 μl DNase (Sigma) and then reverse transcribed with the SuperScript III Reverse Transcriptase protocol (Invitrogen) to obtain cDNA. An amount of 1 μg of cDNA template was used for the qRT-PCR using SYBR Green qPCR Mastermix (Qiagen). Analysis was performed in biological triplicates using expression of GAPDH for normalization of data. The PCR was performed on a 7900HT Fast Real-Time PCR System (Applied Biosystems). The following primers were used:

BRCA1: 5′-TCAACTCCAGACAGATGGGAC-3′; 5′-GGCTGTGGGGTTTCTCAGAT-3′,

GAPDH: 5′-CGAGCCACATCGCTCAGACA-3′; 5′-GGCGCCCAATACGACCAAAT-3′.

### Dose-response curves

Dose–response curves for MMC (Sigma-Aldrich), cisplatin (Sigma-Aldrich), Acetaldehyde (Sigma-Aldrich), Diepoxybutane (Sigma-Aldrich) and Nutlin-3a (Sigma-Aldrich) were performed as biolgical triplicates in 96-well plates by seeding 1000 cells per well, the day before treatment. The following day, drugs were added at 2-fold serial dilutions. Four days after the initiation of the treatment, cell viability was measured using CellTiter-Glo (Promega).

### Colony formation assays

Cells were seeded in 6-well plates the day before the treatment (1000 cells per well). The next day MMC or Nutlin-3a were added at the indicated concentrations. Three days after the initiation of the treatment, drug-containing medium was changed with fresh drug-free medium. Cells were left in culture until visible colonies appeared (7–10 days). Colonies were then fixed in 3.7% formaldehyde in phosphate-buffered saline (PBS) for 1 h, washed in PBS and stained with 0.1% crystal violet solution in PBS supplemented with 10% ethanol for 1 h, followed by washing twice with H_2_O. For quantification, crystal violet was extracted using 50% EtOH and absorbance was measured at 595 nm.

### Remodelin incubation and microscopy

Cells were adhered onto coverslips and incubated with Remodelin at 1 μM for 2 days. To visualize nuclei, cells were subsequently fixed with 4% paraformaldehyde in PBS and stained with 4′,6-diamidino-2-phenylindole (DAPI). Images of cells were acquired using a Deconvolution microscope (Leica). CellProfiler software was used to quantify nuclear circularity and nuclear area from DAPI staining pictures, using the ‘object size shape’ measurement.

### γH2AX staining and analysis

Cells were seeded in three 96-well plates (black with clear flat bottom tissue culture treated imaging microplates from Falcon) and left to adhere over-night. Cells were either treated with 60 nM MMC or left untreated. The experiments were done in triplicate wells. After 24, 48, and 72 h of treatment, cells were fixed with 100% methanol. Staining: Cells were blocked for 1 h with Blocking Buffer (10% FCS and 0.1% Triton X-100 in PBS), incubated for 1 h with the primary antibody (Anti-phospho-Histone H2A.X Ser139, clone JBW301 from Millipore/Upstate) at a dilution of 1:1000 in Blocking Buffer, washed three times with PBS, incubated for 1 h with secondary antibody (Alexa Fluor 488 goat anti-mouse IgG, H+L, from Invitrogen) diluted at 1:600 along with DAPI (0.2 mg/ml stock from Sigma-Aldrich), diluted at 1:1000 in Blocking Buffer, washed 3 times and left in PBS. Cells were imaged on the Operetta-High Content Imaging System (Perkin Elmer, ×20 objective). The image analysis software Cell Profiler was used to quantify the integrated intensity of nuclear γH2AX. Apoptotic cells were excluded from the analysis. For each condition at least 1000 cells were quantified, except for ∆*FANCC* time point 2 and 3 where 877 and 426 cells (respectively) were obtained. R was used for data processing and normalization. The threshold between γH2AX positive (integrated intensity > 10) and negative cells was determined by comparing treated and untreated cells.

### Measurement of apoptosis

Cells were seeded as triplicates on day 1 in 10 cm dishes and treated with 46 nM MMC on day 2, day 3, and day 4. On day 5, cells were stained using the PE Annexin V Apoptosis Detection Kit I from BD Biosciences according to the provided protocol and analyzed by flow cytometry.

### Metaphase spreads

Cells were seeded in 10 cm dishes and treated with MMC for the indicated times. Colcemid (KaryoMAX^TM^, Gibco, Thermo Fisher Scientific) was added at a final concentration of 500 ng/ml 3 h before harvesting. Cells were trypsinized and incubated in KCl 0.075 M (KaryoMAX^TM^, Gibco, Thermo Fisher Scientific) for 6 min. After centrifugation, cells were resuspended in fixation solution (methanol:acetic acid 3:1) and incubated for 15 min at room temperature. Centrifugation and re-suspension in fresh fixation solution was repeated two times. Metaphase spreads, slide preparation and measurement of chromosomal aberrations was performed at Karyologic Inc (North Carolina, USA).

### Immunoblotting and antibodies

Cell extracts were prepared in RIPA lysis buffer (NEB) supplemented with protease inhibitors (Sigma) and phosphatase inhibitors (Sigma, NEB). Immunoblots were performed using standard procedures. Protein samples were separated by SDS–PAGE (4–12% gradient gels; Invitrogen) and subsequently transferred onto nitrocellulose membranes. All primary antibodies were used at 1:1000 dilution with the exception of BLM (1:500) and RMI1 (1:5000). Secondary antibodies were used at 1:5000. The following antibodies were used: FANCC clone 8F3 (Merck Millipore), FANCD2 EPR2302 (Abcam), FANCI A301-254 (Bethyl laboratories), NQO1 clone A180 (Cell Signaling), RMI1 (Proteintech), BLM clone C-18 (Santa Cruz), MUS81 clone MTA30 2G10/3 (Abcam), p21 clone F-5 (Santa Cruz), β-Actin clone 20–33 (Sigma), Tubulin clone DM1A (Cell Signaling), HRP-conjugated goat anti-mouse, rabbit or goat IgG (Jackson Immunochemicals). The p53 antibody (PAB 421) was from Cancer Research UK. Uncropped immunoblot images are shown in Supplementary Fig. 7.

### Genome-wide CRISPR-Cas9 screen

GeCKO CRIPSR library virus was produced as reported^10^ and described briefly following, using both CRISPR library A and library B in one production step: HEK-293T cells were seeded at 40% confluency in T-225 flasks and 24 h later were transfected with GeCKO CRISPR library A and B, pVSVg and psPAX2 plasmids using Lipofectamine® 2000 transfection reagent (Invitrogen, ThermoFisher Scientific) according to the manufacturer's protocol. After 6 h, the medium was changed with DMEM (10% FBS) and after 60 h, virus-containing supernatant was centrifuged at 700 × g at 4°C for 10 min and then filtered through a 0.45 μM filter (Millipore Steriflip HV/PVDF). Cells were infected with a multiplicity of infection (MOI) between 0.3 and 0.5. For each screened cell line, 100 million HAP1 cells were spinfected by centrifugation. Day 1: 12 6-well plates were seeded with 1.5 million cells per well, supplemented with viral supernatant and IMDM (10% FBS, 1% P/S) to reach a volume of 1 ml per well. Polybrene was added at 8 μg/ml. Cells were spinfected for 3 h at 724×*g* at 37 °C, pooled and transferred into 15 cm dishes. Day 3: Cells were challenged with 2 μg/ml puromycin to deplete uninfected cells. Day 5: WT cells were challenged with 30 ml of 190 nM mitomycin C (MMC) in IMDM (10% FBS, 1% P/S) per 15 cm dish. ∆*FANCC* cells were challenged with 30 ml of 46 nM MMC in IMDM (10% FBS, 1% P/S) per 15 cm dish. Treated cells were incubated for 10 days following MMC challenge, WT untreated cells were split every 2–3 days for 10 days to avoid confluency, re-seeding > 100 million cells each time. Genomic DNA of at least 30 million cells per sample was extracted using the Qiagen Blood & Cell Culture DNA Maxi Kit according to the manufacture’s protocol. PCR was performed in two steps, using PCR1- and barcoded PCR2 primers as reported^10^, obtained from http://genome-engineering.org/gecko/wp-content/uploads/2013/12/GeCKO-plasmid-readout-primers-July2014.xlsx. PCR1 amplified the gRNA sequences of 130 μg genomic DNA in 13 × 100 μl reactions per sample using the Promega GoTaq^®^ G2 DNA Polymerase. PCR1 reaction tubes were pooled for each sample. PCR2 added Illumina sequencing adapters by performing 16 × 100 μl PCR reactions per sample with 2 μl input DNA from PCR1 per reaction tube. PCR program for PCR1 and PCR2: Heat lid 110 °C; 94 °C 2 min; loop 18× (94 °C 30 s; 55 °C 30 s; 68 °C 1 min) 68 °C 7 min. PCR2 products were purified by running them on agarose gel and DNA was extracted using the Promega Wizard^®^ SV Gel and PCR Clean-Up System. Barcoded samples were pooled and submitted to the Biomedical Sequencing Facility (BSF) for 61 base pair single-end sequencing. Barcoded samples from the CRIPSR library screen were de-multiplexed by the BSF. Enrichment analysis for gRNAs was performed using the MAGeCK-VISPR analysis and visualization software^36^ by comparing the MMC treated *∆FANCC* sample to WT untreated, or WT MMC treated to WT untreated respectively (positive selection).

### Genome-wide insertional mutagenesis screen

For gene-trap insertional mutagenesis we followed the published protocol^5^ as described briefly following: Gene-trap virus was produced in HEK293T, that were seeded in 15 cm dishes and transfected with the gene-trap plasmid and packaging plasmids VSVg, gag-pol and pAdVAntage™ Vector (Promega) using Lipofectamine® 2000 Transfection reagent (Invitrogen, ThermoFisher Scientific) according to manufecturer’s protocol. The following day, medium was replaced with fresh DMEM (20% FBS, 1% Pen/strep). Retroviral supernatant was collected for three consecutive days, centrifuged at 700 × g for 10 min, filtered through a 0.45 μM filter (Millipore Steriflip HV/PVDF) and ultracentrifuged at 70,737 × g (average RCF) at 4 °C for 90 min with a SW 32Ti rotor (Beckman Coulter). Viral pellets were re-suspended in PBS, pooled and *ΔFANCC* HAP1 cells were transduced with concentrated retrovirus containing the gene-trap cassette^5^. After integration of the GFP-expressing gene-trap cassette, cells were analyzed by flow cytometry to measure efficiency of infection and populations with > 70% GFP-expressing cells were used for treatment with MMC. The control non-selected WT HAP1 data-set was taken from Blomen et al.^8^. 100 million cells from the mutagenized pools were seeded in 15 cm dishes at a density of 6 million cells per dish. The following day MMC was added at a concentration that selectively killed FANCC-deficient cells, leaving only around 5–10% of cells surviving (46 nM for FANCC). Cells were left to grow for 10 days. After the end of treatment cells were trypsinized and frozen at −80 °C. For preparation of the gene-trapped DNA libraries, genomic DNA was extracted from 30 million cells using QIAamp DNA mini kit (Qiagen), subjected to digestion with MseI (NEB) and NlaIII enzymes (NEB) and subsequently ligated by T4 DNA ligase (NEB). Digested and ligated fragments were used as template for inverse PCR with primers targeting the LTR regions of the gene-trap cassette. After amplification and purification of the fragments the DNA sample was submitted for next generation sequencing (Illumina HiSeq 2000, 50 base pair single-read) to the CeMM Biomedical Sequencing Facility (BSF). Bioinformatics analysis of the next generation sequencing data was done in R according to Carette et al.^5^ as follows: Briefly, raw sequencing data was aligned to human reference genome hg19 (UCSC hg19 build) using bowtie2 (version 2.2.4) with default parameter. Reads were removed that did not meet the following criteria: (1) have a reported alignment-“mapped reads” (2) have a unique alignment (3) have a mapping quality (MAPQ) higher than 20. Duplicate reads were marked and discarded with Picard (version 1.111). Insertions in close proximity (1 or 2 base pairs distance from each other) were removed to avoid inclusion of insertions due to mapping errors. Insertions were annotated with gene build GRCh37.p13 (ENSEMBL 75-release February 2014) using bedtools (version 2.10.1) and custom scripts. The canonical transcripts (according to ENSEMBL) for each gene were used as a reference gene model to count insertions falling with exons, introns or intragenic. Insertions were considered mutagenic or disruptive to the gene if they occurred within exons irrespective of their orientation to the corresponding gene or if they were located within introns in sense orientation. Insertions in antisense direction in respect to the gene orientation were considered silent. All mutagenic insertions were summarized independently for each gene. For each gene a one-sided Fisher’s exact-test was applied to estimate a significant enrichment of insertions over an unselected control data set.

### Statistical analysis

For gene-trap and CRISPR library screens, hit selection was performed in two steps. First, each data set was partitioned into two groups, defining the hit-group as data points with *p* < 0.001 and fold-change > 2^1.5^. In the second step, hit selection was optimized using linear discriminant function analysis. Dose-points of survival curves indicate the mean of biological triplicates, with S.E.M. shown as error bars. The *p*-values for the γH2AX staining were determined by two-way ANOVA. Means and S.E.M. of biological triplicates are plotted. The *p*-values for the chromosomal breaks and gaps/metaphase were determined by Mann–Whitney U test. Means and S.E.M. of biological triplicates are plotted.

### Data Availability

All data generated or analyzed during this study is included in this published article and its Supplementary Information.

## Electronic supplementary material

Supplementary Information

Peer Review File
