# Supplementary material for: Parallel genome-wide screens identify synthetic viable interactions between the BLM helicase complex and Fanconi anemia
Source: Nat Commun. 2017 Nov 1;8:1238. doi: 10.1038/s41467-017-01439-x (PMC5663702; doi:10.1038/s41467-017-01439-x)
Supplement: Supplementary file 1 — Supplementary Information [file 41467_2017_1439_MOESM1_ESM.pdf]

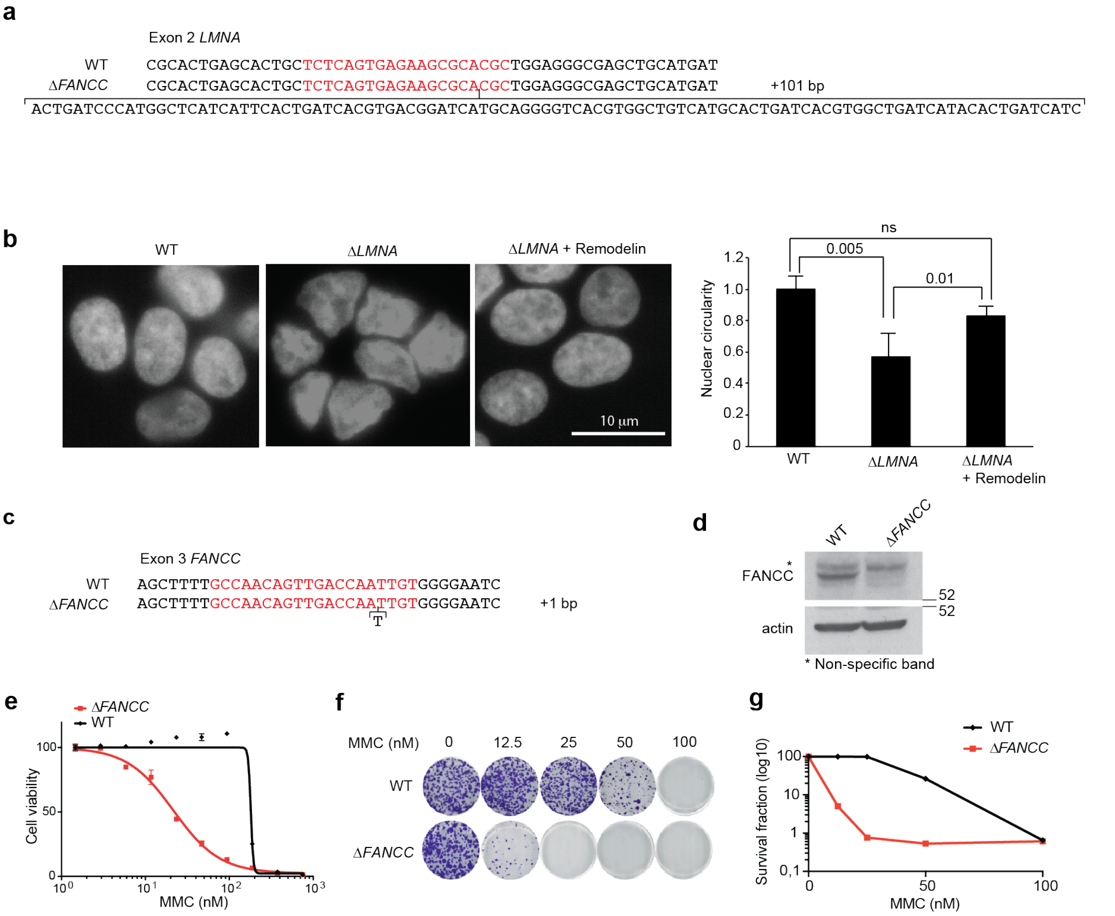

Supplementary Figure 1 Characterization of HAP1 cells for synthetic viable interactions and generation of Δ*FANCC* cells. (a) CRISPR-Cas9-mediated mutation of lamin A in human HAP1 cells (Δ*LMNA*). The red sequence in WT corresponds to the gRNA used. (b) DAPI staining of WT and Δ*LMNA* cells with or without incubation with the NAT10 inhibitor Remodelin, with quantification of nuclear circularity. (c) CRISPR-Cas9-mediated mutation of *FANCC* in human HAP1 cells (Δ*FANCC*). The red sequence in WT corresponds to the gRNA used. (d) Immunoblot of WT and Δ*FANCC* cell extracts for FANCC and actin. * denotes a non-specific band. (e) Survival of WT and Δ*FANCC* cells following MMC exposure for 4 days, assessed by CellTiter-Glo. Means and S.E.M. of triplicates are plotted. (f) Colony formation of WT and Δ*FANCC* cells following exposure to MMC for 10 days. (g) Quantification of colony formation shown in (f).

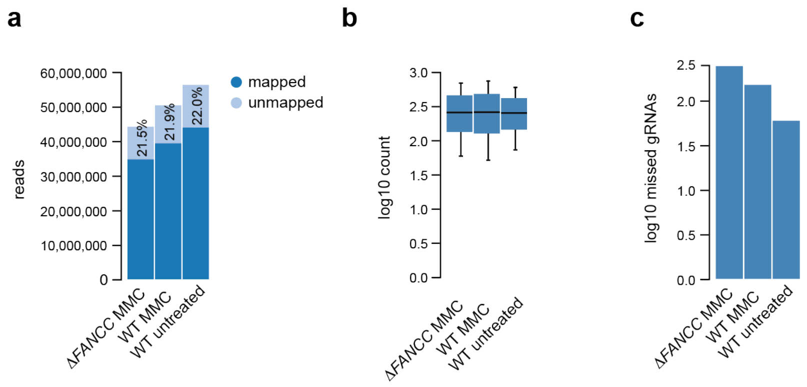

Supplementary Figure 2. Quality control of genome-wide CRISPR library and insertional mutagenesis. (a) ‘Reads’ depicts the total number of sequenced reads for each sequenced library. (b) ‘Log10 count’ indicates the average amount of sequenced reads for each gRNA. (c) ‘Log 10 missed gRNAs’ shows the average amount of gRNAs missing in the sequenced samples compared to the total list of gRNAs.

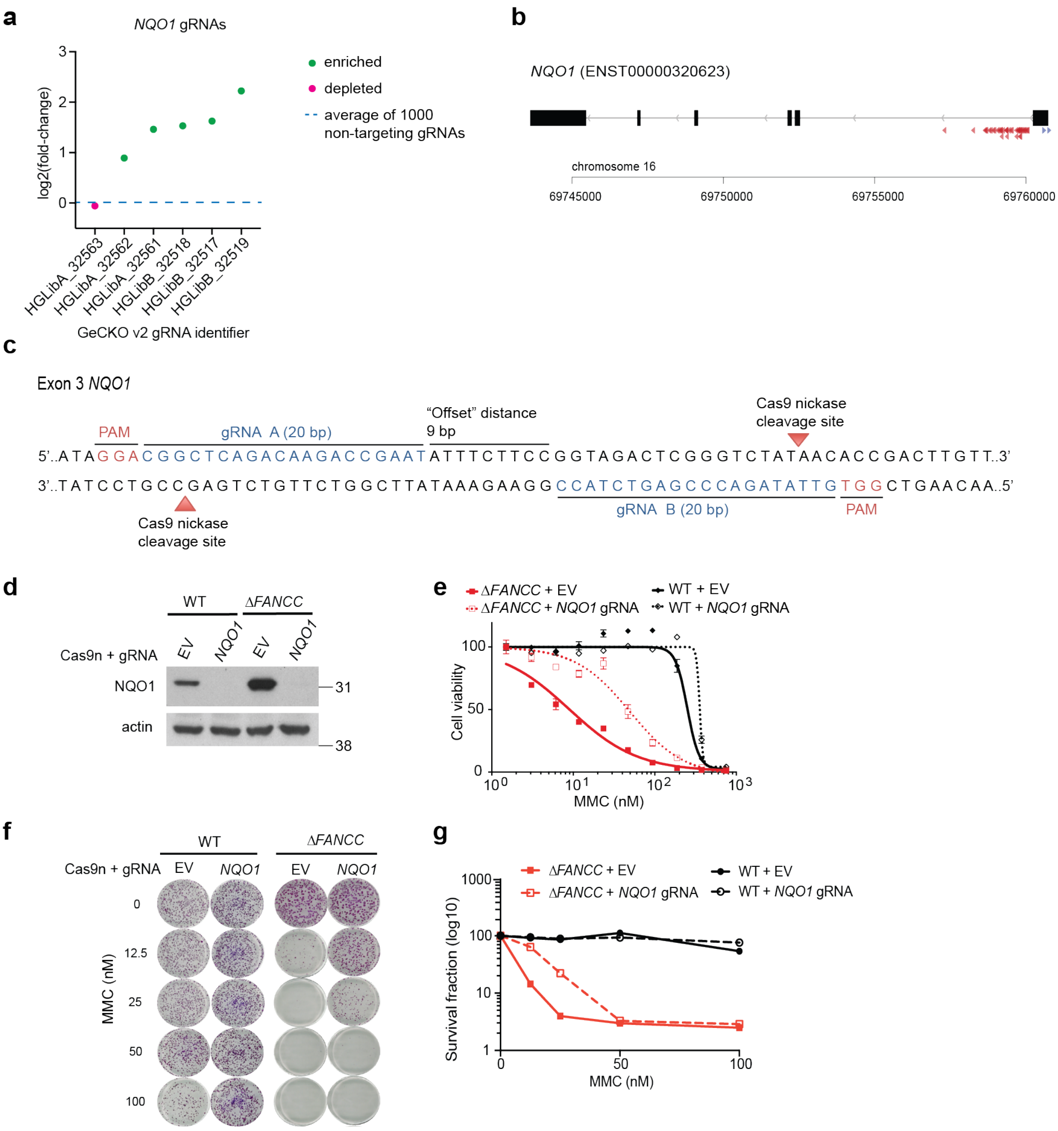

Supplementary Figure 3. Validation of NQO1 as a suppressor gene following exposure to MMC. (a) Enriched gRNAs (5 out of 6) for *NQO1* obtained in the CRISPR screen by treating Δ*FANCC* cells with MMC, compared to WT untreated cells. (b) Insertion sites (42) within *NQO1* obtained in Δ*FANCC* cells treated with MMC. Red arrows indicate insertions in the sense orientation (inactivating in both intronic and exonic regions, n=40) while blue arrows indicate insertions in the antisense orientation (inactivating only in exonic regions, n=2). (c) Sequences and position of the gRNA pair selected to target *NQO1* using Cas9 nickase. (d) Immunoblot for NQO1 expression in cell extracts obtained from WT or Δ*FANCC* cells either infected with an empty vector (EV) or with a vector expressing gRNAs and nickase Cas9 ('Cas9n + gRNA'). (e) Survival of WT and Δ*FANCC* cells transduced with an empty vector (EV) control plasmid ('WT + EV' and 'Δ*FANCC* + EV') or with a plasmid expressing gRNAs targeting *NQO1*, along with nickase Cas9 ('WT + *NQO1* gRNA' and 'Δ*FANCC* + *NQO1* gRNA'), following MMC exposure for 3 days, assessed by CellTiter-Glo. Means and S.E.M. of triplicates are plotted. (f) Colony formation assay of cells indicated in (e) following MMC exposure for 10 days. (g) Quantification of colony formation shown in (f).

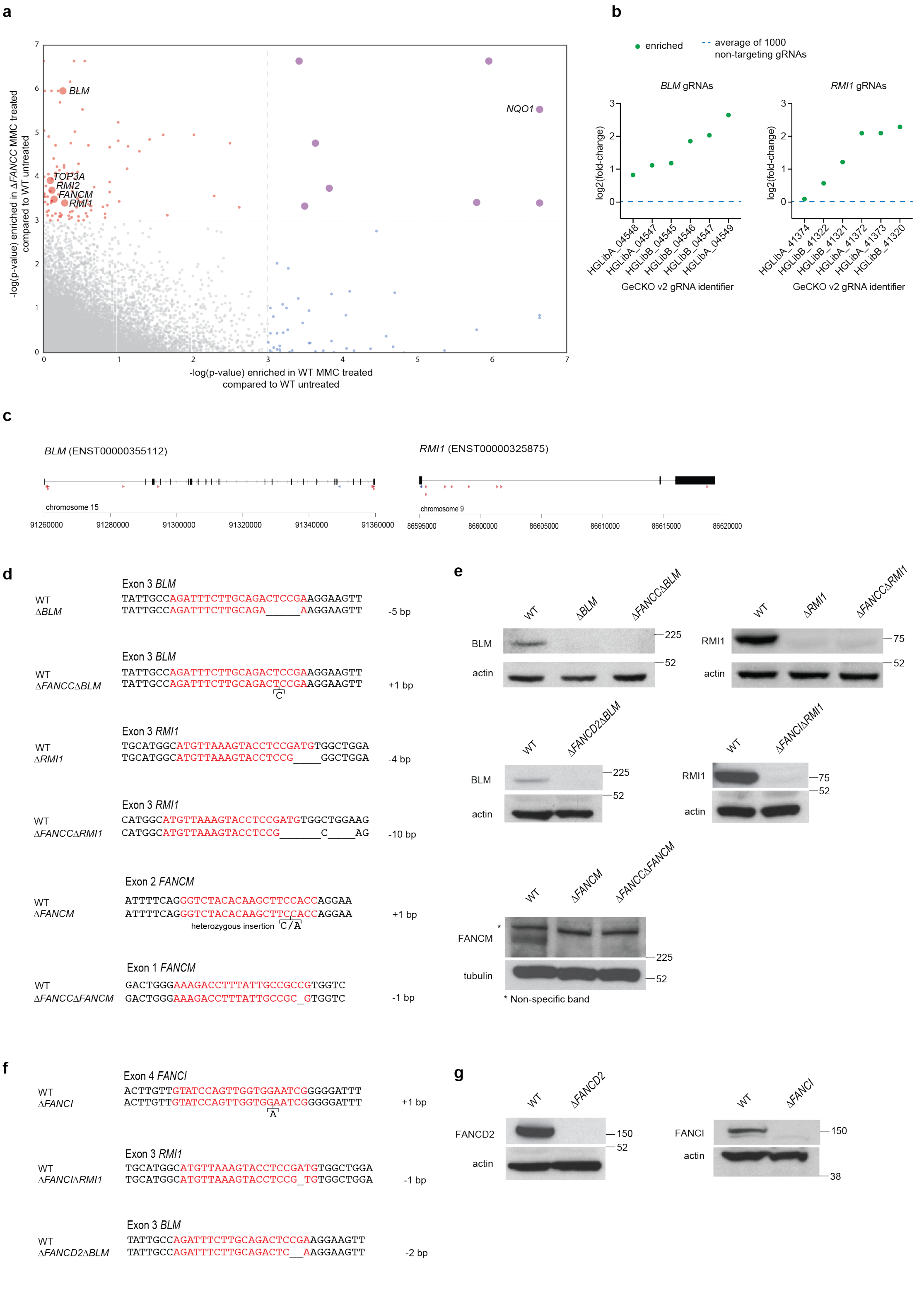

Supplementary Figure 4. Validation of the BLM complex as a suppressor for FA mutant cells following exposure to MMC. (a) Mutated genes enriched in MMC treated Δ*FANCC* cells, compared to untreated WT cells, plotted against mutated genes enriched in MMC treated WT cells, compared to untreated WT cells, according to p-values from the CRISPR screen. (b) Enriched gRNAs in the CRISPR screen for BLM and RMI1 in Δ*FANCC* cells treated with MMC compared to WT untreated cells.

(c) Gene-trap insertions within *BLM* and *RMI1* enriched in Δ*FANCC* cells treated with MMC. Red arrows indicate mutagenic insertions in the sense orientation (10 and 9 unique inactivating insertion sites for *BLM* and *RMI1* respectively) while blue arrows indicate insertions in the antisense orientation (inactivating only in exonic regions; 1 identified for both *BLM* and *RMI1*). (d) CRISPR-Cas9-mediated mutation of *BLM, RMI1* and *FANCM* in WT HAP1 cells or in Δ*FANCC* mutant cells. Red sequences in WT correspond to the gRNAs used. (e) Immunoblots of BLM, RMI1, FANCM and actin from cell extracts of indicated cells. (f) CRISPR-Cas9-mediated mutation of *FANCI* in WT cells, *RMI1* in Δ*FANCI* cells and *BLM* in Δ*FANCD2* cells. Red sequences in WT correspond to the gRNAs used. (g) Immunoblots of FANCD2, FANCI and actin from cell extracts of indicated cells.

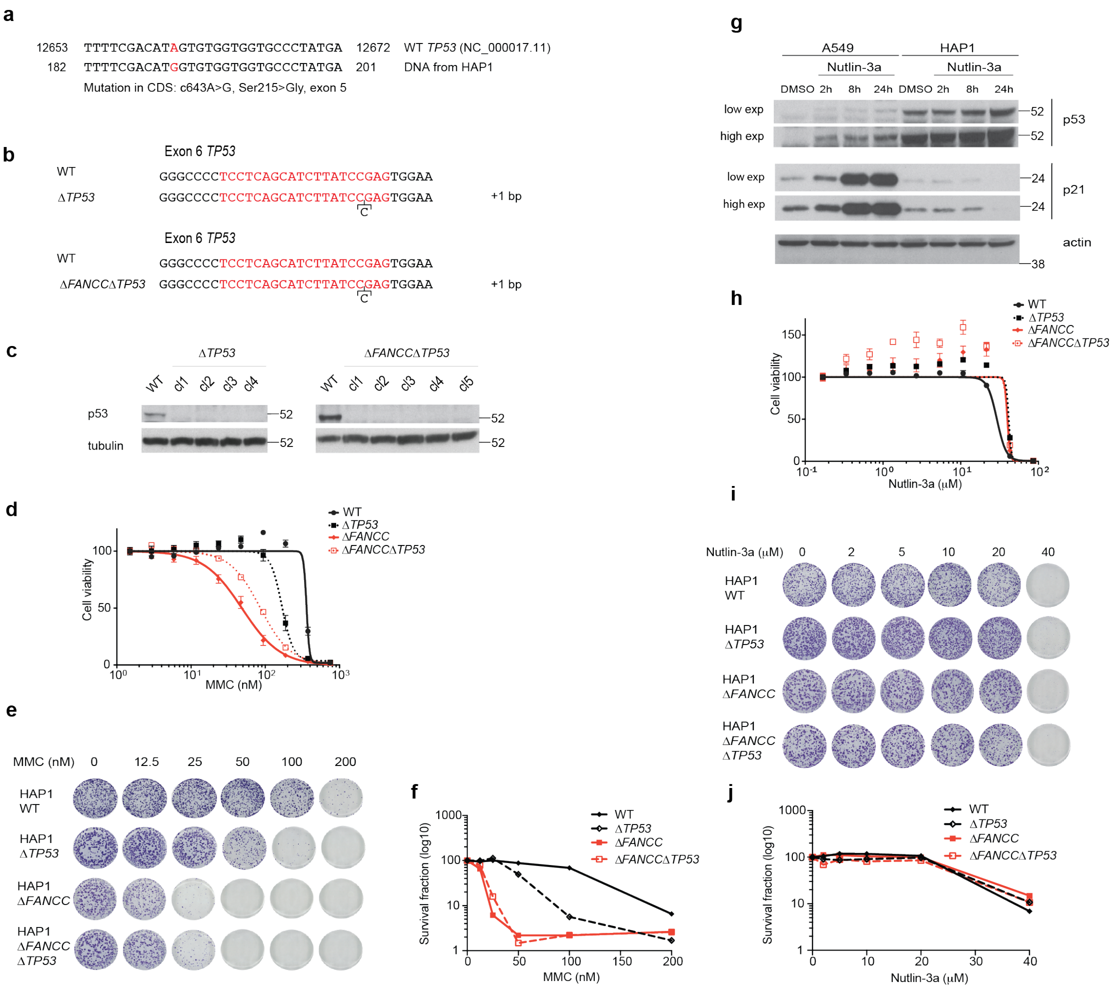

Supplementary Figure 5. Characterization of p53 in HAP1 cells. (a) Mutation of the *TP53* gene in HAP1. (b) Targeting of *TP53* by CRISPR-Cas9 in WT and Δ*FANCC* cells. Sequences of *TP53* in Δ*TP53* and Δ*FANCC*Δ*TP53* indicate mutations in clone 3 (cl3; see panel 'c') in both cell lines. (c) Immunoblot of p53 knock-out clones in WT and Δ*FANCC* deficient cells. Clone 3 (cl3) of cell lines was chosen for further experiments. (d) Survival of indicated cells after treatment with MMC. Means and S.E.M. of triplicates are plotted. (e) Colony formation assay of indicated cells exposed to MMC. (f) Quantification of (e). (g) Immunoblot of A549 and HAP1 cells exposed to Nutlin-3a at 10 μM for the indicated time points and probed for p53, p21 and actin. (h) Survival of indicated cells after Nutlin-3a treatment. Means and S.E.M. of triplicates are plotted. (i) Colony formation assay of indicated cells to Nutlin-3a. (j) Quantification of (i).

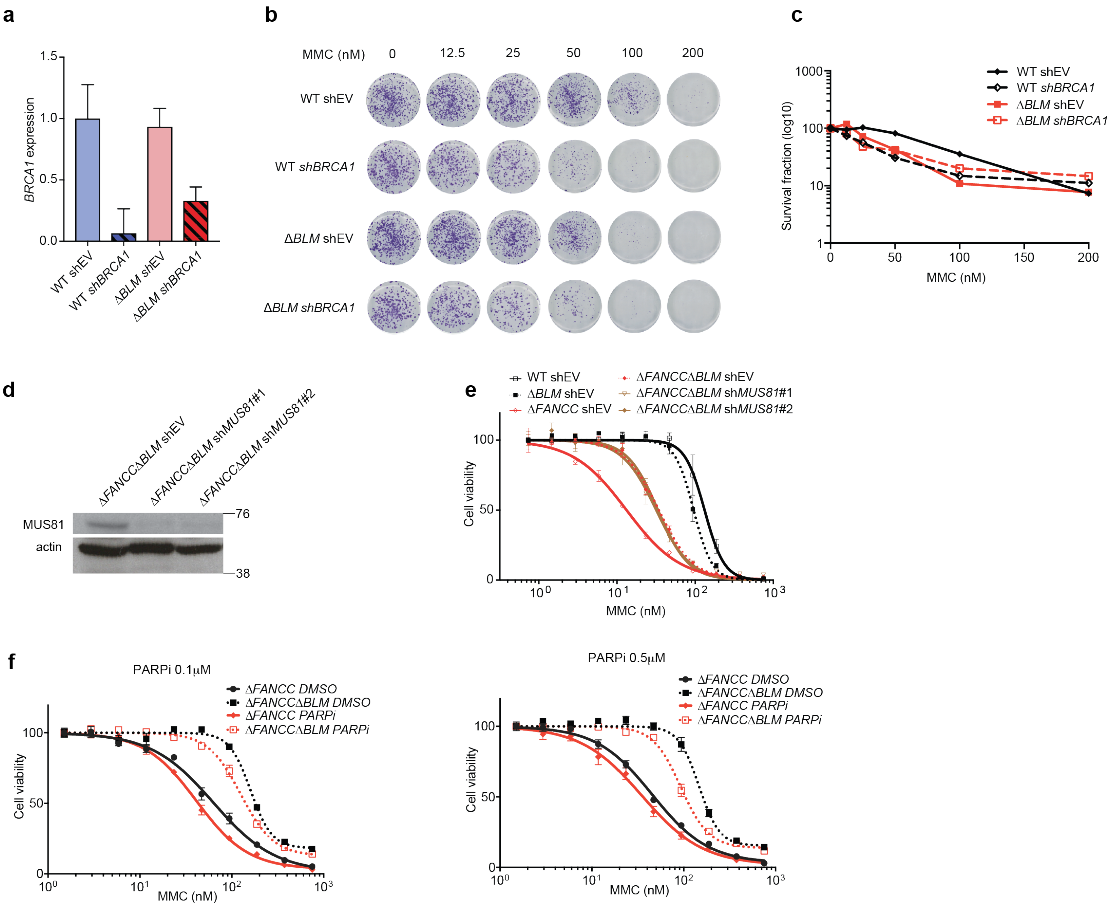

Supplementary Figure 6. Mechanisms of rescue in Δ*FANCC*Δ*BLM* cells. (a) Expression of *BRCA1* upon shRNA knock-down measured by quantitative reverse transcription PCR (q RT-PCR) in WT and Δ*BLM* cells compared to cells infected with empty vector (EV). (b) Colony formation assay of WT and Δ*BLM* cells infected with sh*BRCA1* or shEV treated with MMC. (c) Quantification of (b). (d) Immunoblot for MUS81 knock-down in Δ*FANCC*Δ*BLM* cells using two different shRNAs (sh*MUS81*#1, sh*MUS81*#2), compared to Δ*FANCC*Δ*BLM* cells infected with empty vector (shEV). (e) Survival of indicated cells infected with shEV, sh*MUS81*#1 or sh*MUS81*#2, treated with MMC, assessed after 4 days by CellTiter-Glo. (f) Survival of Δ*FANCC* or Δ*FANCC*Δ*BLM* cells treated with either the PARP inhibitor olaparib (PARPi) or DMSO for 4 hours, followed by MMC exposure for four days, assessed by CellTiter-Glo. Error bars of survival curves and expression data indicate S.E.M. of triplicates.

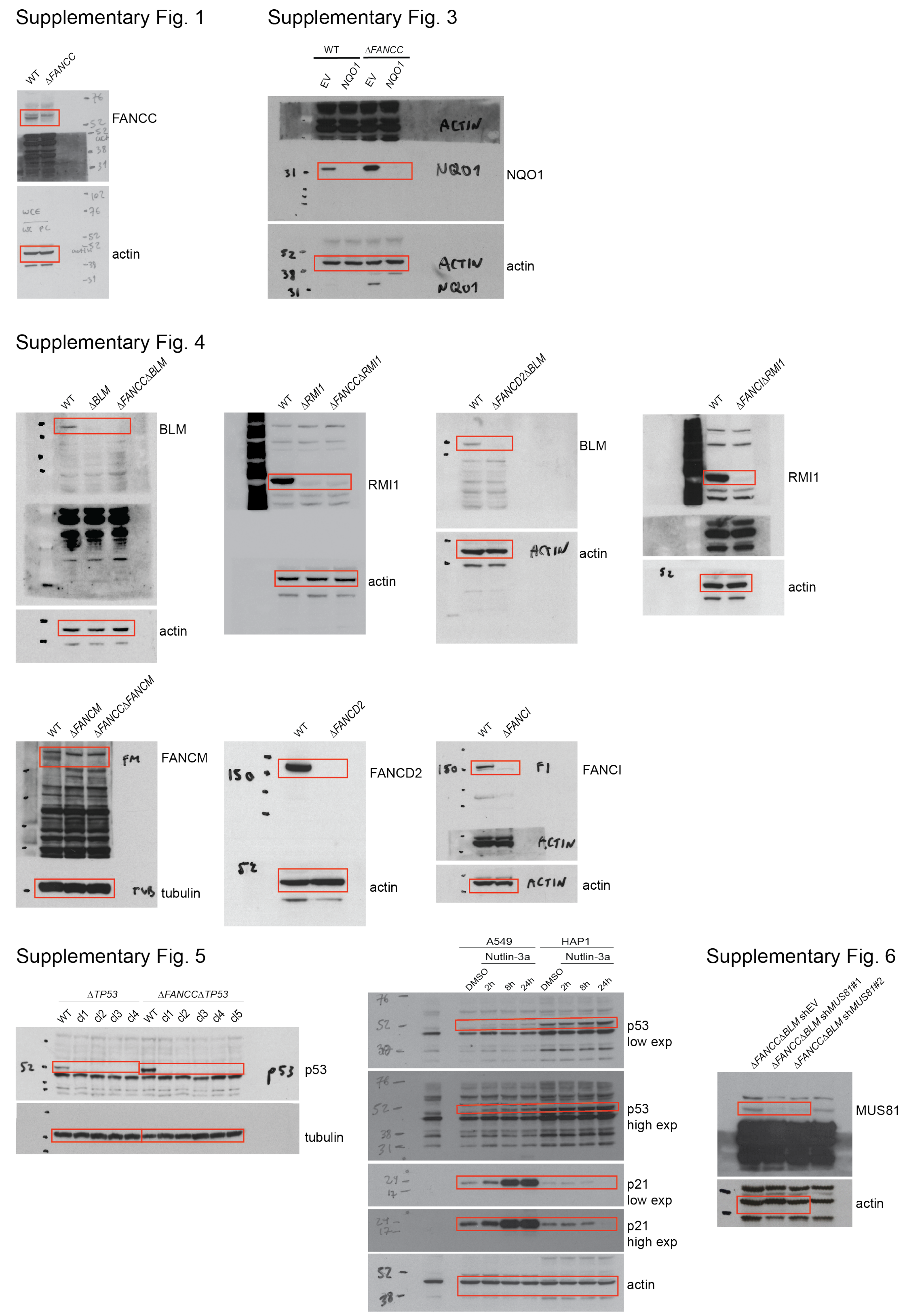

Supplementary Figure 7. Uncropped immunoblots.

Sample	Number of genes targeted*	Total number of insertions
HAP1 Δ*FANCC* + MMC	7,236	22,772
HAP1 WT untreated	17,907	2,274,503

* protein-coding

Supplementary Table 1. Table depicting the number of genes targeted and the total number of unique insertions for each library sequenced using the insertional mutagenesis gene-trap approach.

		Viable		Late apoptosis		Early apoptosis	
	**Time point**	**Mean**	**S.E.M.**	**Mean**	**S.E.M.**	**Mean**	**S.E.M.**
**Δ*WT***	**UT**	86.0	1.7	3.8	0.7	10.2	1.0
	**24h**	90.0	0.6	2.7	0.3	7.3	0.4
	**48h**	88.1	0.3	6.0	0.7	6.0	0.9
	**72h**	66.1	1.3	22.6	1.8	11.3	0.6
**Δ*BLM***	**UT**	81.4	0.6	6.0	1.0	12.6	1.4
	**24h**	87.7	0.8	4.3	0.4	8.0	0.5
	**48h**	78.7	1.9	12.8	1.6	8.5	0.3
	**72h**	62.1	2.4	26.5	1.7	11.4	1.1
**Δ*FANCC***	**UT**	71.3	1.7	3.5	0.1	25.3	1.8
	**24h**	68.9	0.5	4.6	0.7	26.5	0.6
	**48h**	37.4	1.3	33.1	0.6	29.5	1.9
	**72h**	15.4	1.1	63.6	1.7	21.1	0.7
**Δ*FANCC*Δ*BLM***	**UT**	80.0	1.9	4.8	0.5	15.2	2.0
	**24h**	81.0	0.9	4.8	0.7	14.2	1.5
	**48h**	74.2	1.2	11.6	0.8	14.2	1.6
	**72h**	41.1	1.8	37.4	3.2	21.5	1.9

Supplementary Table 2. Table numerically showing the percentage of cells that are viable, in late or early apoptosis as graphically depicted in Fig. 2e.
