# Supplementary material for: Parallel genome-wide screens identify synthetic viable interactions between the BLM helicase complex and Fanconi anemia
Source: Nat Commun. 2017 Nov 1;8:1238. doi: 10.1038/s41467-017-01439-x (PMC5663702; doi:10.1038/s41467-017-01439-x)
Supplement: Supplementary file 2 — Peer Review File [file 41467_2017_1439_MOESM2_ESM.pdf]

Reviewers' Comments:

Reviewer #1 (Remarks to the Author)

The manuscript "Parallel genome-wide screens identify a synthetic viable interaction between the BLM helicase complex and Fanconi anemia" by Moder et al. uses CRISPR and insertional mutagenesis technologies in haploid cells to identify genes that, when deleted, suppress the known hypersensitivity of FANCC-deficient cells to the interstrand crosslinking agent, mitomycin C (MMC). Results suggest that loss of function of members of the BLM helicase/dissolvase complex (specifically BLM, RMI1, RMI2, and TOP3A) suppress the MMC hypersensitivity of FANCC (and FANCD2)-deficient cells. While the methodologies used are relatively innovative and the data presented intriguing, several issues detailed below detract from the manuscript in its current form.

Specific comments

1. (lines 86-92, Fig. S1) While this data reveals that the investigators can recapitulate a synthetic viable relationship shown previously using a chemical inhibitor on a cell line with a directed gene inactivation, this example is not analogous to the situation described in the remainder of the study--i.e., identification of genes that when disrupted, suppress a phenotype caused by another inactivated gene. As such, this example is not particularly valuable as support for the remainder of the study and could be removed. Furthermore in regard to this section, since lamin A is primarily a nuclear envelope factor and not a DNA repair gene, the phrase "to validate the use of HAP1 as a cellular model system in which to identify genetic synthetic viable interactions for DNA repair genes" (lines 86-87) is misleading. In fact, this reviewer has some concern about the use of haploid cells to examine phenotypes (including rescue of MMC sensitivity) that might be affected by HR pathways, due to the reality that HR between homologous chromosomes is not possible in haploid cells as compared with diploid cells. To alleviate any concern about the specificity of the relationship between the FA and BLM pathways, the authors could perform a parallel study in a standard diploid FANCC-deficient cell line to knockdown or knockout BLM (or RMI1, RMI2, or TOP3A) and confirm resistance to MMC specifically in the doubly-deficient condition.

2. (Fig. 1) The evidence showing FANCC targeting and knockout is not very convincing. The Western blot (Fig. 1b) showing FANCC expression is suboptimal due to the low level of signal even in the wild type lane (the same issue exists for knockout of FANCI expression in Fig,. S4g). Either this blot needs to be overexposed or redone so that the control is very prominent and the knockout is completely absent under better exposure conditions. In addition or alternatively, specificity for FANCC deficiency in the viability and colony assays could be strengthened by showing that transfection of wild type FANCC into FANCC cells restores survival after MMC treatment. Also, authors should show a graphical depiction of their colony forming assay results.

3. While the data presented are intriguing, the endpoint of viability used by the authors is a relatively crude measure of linkages between pathways. A molecular linkage between the FANC and BLM pathways that both impact genome stability would be significantly strengthened by additional experimentation that confirms that deficiency in a BLM complex component rescues the DNA metabolic defect caused by FANC deficiency. For example, does inactivation of BLM complex components help facilitate ICL removal, suppress MMC-induced chromosome aberrations (radial formation) in FANC cells, or suppress double-strand break formation during ICL repair (as suggested in lines 166-168)?

4. (lines 151-171) The authors should more clearly develop and discuss their ideas about how loss of the BLM complex rescues MMC hypersensitivity caused by deficiency of FANCC or other FA components. In this regard, there is a significant amount of literature linking the BLM and FA

pathways, including reports showing physical interaction of BLM with FANC components; very little of this literature is mentioned in the authors' brief treatment here. Also, some of the results suggest near complete suppression of MMC hypersensitivity; it seems highly unlikely that loss of an HR pathway would restore "normalcy" in survival to cells that lack the ICL repair pathway dependent on HR. In regards to specifying the effect of the observed suppression, the authors should also consider the possibility that the BLM dissolvasome complex may be required to relieve topological strain between replication forks converging upon an ICL, an activity potentially necessary to yield a relevant DNA structural intermediate early in crosslink repair. By this reasoning, loss of BLM complex components could thus channel ICL repair into an FA-independent pathway.

Minor comments

1. (p. 2, lines 60-74) The authors should clearly define synthetic viability here as the suppression of a genetic defect or phenotype by abrogation of another gene or pathway. Moreover, although the term "synthetic viability" has been used previously, this reviewer feels this is somewhat misleading as the strategy should hopefully suppress the effects of a metabolic defect even under conditions where the affected cell or organism retains viability. Instead, this strategy better reflects identification of "synthetic" suppressors.

2. (Fig. 2c, d) If NQO1 is required for activation of MMC to generate ICLs, clarify why the survival of delta FANCC-deltaNQO1 cells in response to MMC is not identical to wild type cells treated with MMC.

3. (lines 147-149) The statement "Following exposure to MMC, we observed …" suggests a lack of 'rescue' of WT cells by loss of BLM or RMI1 when these cells are not MMC hypersensitive in the first place and show essentially no change. This statement should be reworded for clarification.

4. (lines 168-171) This statement appears to be backward. Specifically, depletion of HR factors suppresses deficiencies in the Sgs1-Top3 complex, according to data from the cited reference. Thus, this concept may be less relevant to the scenario described in the current study.

5. References #13 and #23 are incomplete.

Reviewer #2 (Remarks to the Author)

In this interesting paper Loizou et carries out a genome wide synthetic viability screen to look for genes that confer sensitivity to the DNA crosslinking agent MMC to human cells carrying the FA DNA repair activity. The base of of their screen is the haploid CML cell line HAP1 which has been the focus on many recent genetic greens by virtue of the lines haploid genome. Using this approach they pull out an established target - NQO but they also pull out several genes that function in the Blm complex (Blm being mutated in Bloom syndrome and involved in HJ junction resolution). They further validate these targets and also show that this synthetic viability can also be extended to other FA genes particularly those that work downstream of FANCC such as FANCD2 and FANCI. Whilst this is certainly an interesting and potentially important finding it is my view that the analysis and implications of this are much underdeveloped to merit publication in its current form.

1. Te authors finding is not replicated in the chicken B cell line DT40. See EMBO J. 2005 Jan 26; 24(2): 418–427. This is rather intriguing and may argue that the discovery reported here may be specific to HAP1 and possibly due to a haploid genome.

2. Whilst the authors see that whilst Blm deletion suppresses MMC sensitivity in FA mutant HAP1 cells they present no evidence why this suppression occurs. For instance is chromosome breakage mitigated ? is Apoptosis to DNA damage been attenuated ? In addition dose this also suppress

sensitivity to other crosslinking agents such as Cis-platin and DEB. In addition the possible physiological source of DNA cx might be the aldehydes - formaldehyde and formaldehyde ?

3. In many cellular and animal systems p53 deletion suppresses the sensitivity of FA repair mutant cells lines to crosslinking agents. It is therefore a surprise that the authors to not pick up p53 or its effectors. This could mean that Hap1 might be defective in p53or indeed in its p53 cell cycle and apoptosis responses. The authors really ought to clarify this, and this is important as knowing the functional p53 status of Hap1 has implications for the many people who use this system. In the end if this cell line has a defect in this and since we know that p53 has such a huge impact on the viability of cells to DNA damage then this fact needs to be established before one draws conclusions about DNA damage impact on cell viability.

4. The FANCM gene is part of the FA complex but is also part of the Blm complex. Some functional evidence links the FA pathway to Blm through this interaction. have the authors deleted FANCM if so does this suppress sensitivity ? This is an important point to clarify as the original FANCM patient in fact had a mutation in FANCA and strikingly was only mildly effected - this work here may provide a possible mechanism for this. Indeed see Nat Struct Mol Biol 12 (9), 763-771 here FANCM deletion combined with FANCC suppresses Cx sensitivity.

5. I do not think that blocking Blm is a plausible way to treat FA - Bloom syndrome is much worse illness than FA !In any case point 1 needs to addressed to determine how this suppression works is because DNA damage does not persist or that the response to DNA damage is impacted that also means that point 3 needs to be addressed

Reviewer #3 (Remarks to the Author)

The manuscript by Moder et al. reports the use of CRSIPR and insertional mutagenesis screens in haploid cell lines to identify synthetic viable interactions with deletion of FANCC, a protein of the Fanconi anaemia pathway. The screening protocol described in the paper is robust and certainly will be of interest to the Fanconi and DNA damage response field. The authors first establish the suitability of HAP1 cells for synthetic viable interactions with DNA repair factors by recapitulating previously reported remodelin (NAT10 inhibitor) reversal of ageing phenotypes in cells lacking lamin A. Then, they identify a novel and exciting synthetic viable interaction between FANCC and BLM in response to MMC treatment. Two separate approaches are used for screening: CRISPR/Cas9 genome-wide library and insertional mutagenesis. Both led to identification of NQO1, a reductase previously shown to rescue MMC-induced lethality in Fanconi cells. Furthermore, deletion of BLM interacting partners RMI1, RMI2 or TOP3A can reverse MMC toxicity in FANCA-deleted cells. Thus, antagonism between the Fanconi pathway and BLM complex in the repair of MMC-induced damage is clearly demonstrated and will help characterise mechanisms of drug resistance informative to the clinic.

The problem however, is that the paper lacks mechanistic insight, apart from two speculative scenarios described in Discussion. Moreover, as the paper focuses on a genetic screen, the top hits obtained from the screen should be reported in full, as it is customary for this type of papers. Therefore, I am not sure that Nature Communication is the most suitable journal for publication of this work.

Specific comments:
Introduction:
p.2 Line 61: It should be further explained that cancers in FA patients cannot be treated with conventional chemotherapy, such as platinum drugs due to their increased sensitivity.
Results:
p.4 line 121: Cells showed reduced toxicity, but not resistance in Figure 2d
p. 4 line 122: It should be explained in more detail that NQO1 does not represent a true “synthetic viable” interaction with the FA pathway. Pathologies seen in FA patients mostly arise from their

inability to resolve endogenously formed ICLs or stalled replication forks. Inactivation of NQO1 however is only able to rescue FA-deficient cells, which are treated with the exogenous agent MMC, therefore this interaction may not be physiologically relevant.
p. 4 line 123: The complete list of synthetic viable interactions, which were identified in the two screens described, should be given.
p. 4. line 144: BLM or RMI1 could rescue "cells lacking" FANCI and FANCD2

Discussion:
The authors hypothesize that the absence of the BLM complex leads to preferential resolution of ICLs by structure specific nucleases (MUS81 or GEN1), which promotes survival of FA-pathway deficient cells. The authors could test this by depleting MUS81 or GEN1 in cells lacking FANCA and BLM, and analyse if depletion of these factors re-sensitizes cells to MMC.
Furthermore, the authors hypothesize that BLM contributes to the formation of double strand breaks, which requires the HR for their repair. It is therefore conceivable that BLM inactivation also rescues cells lacking HR factors, such as BRCA2. This could be tested in clonogenic survival assays.
Furthermore, it would be interesting to test whether BLM inhibition using chemical inhibitors (eg. ML261) could recapitulate the synthetic viable interaction between BLM and FANCC.

Reviewer #1 (Remarks to the Author):

The manuscript "Parallel genome-wide screens identify a synthetic viable interaction between the BLM helicase complex and Fanconi anemia" by Moder et al. uses CRISPR and insertional mutagenesis technologies in haploid cells to identify genes that, when deleted, suppress the known hypersensitivity of FANCC-deficient cells to the interstrand crosslinking agent, mitomycin C (MMC). Results suggest that loss of function of members of the BLM helicase/ dissolvase complex (specifically BLM, RMI1, RMI2, and TOP3A) suppress the MMC hypersensitivity of FANCC (and FANCD2)-deficient cells. While the methodologies used are relatively innovative and the data presented intriguing, several issues detailed below detract from the manuscript in its current form.

We thank this reviewer for their positive remarks with regard to innovation. Furthermore, we are pleased that the reviewer found our data to be intriguing and for making several suggestions that have been addressed in full and indeed have improved the manuscript.

Specific comments

1. (lines 86-92, Fig. S1) While this data reveals that the investigators can recapitulate a synthetic viable relationship shown previously using a chemical inhibitor on a cell line with a directed gene inactivation, this example is not analogous to the situation described in the remainder of the study--i.e., identification of genes that when disrupted, suppress a phenotype caused by another inactivated gene. As such, this example is not particularly valuable as support for the remainder of the study and could be removed. Furthermore in regard to this section, since lamin A is primarily a

nuclear envelope factor and not a DNA repair gene, the phrase "to validate the use of HAP1 as a cellular model system in which to identify genetic synthetic viable interactions for DNA repair genes" (lines 86-87) is misleading. In fact, this reviewer has some concern about the use of haploid cells to examine phenotypes (including rescue of MMC sensitivity) that might be affected by HR pathways, due to the reality that HR between homologous chromosomes is not possible in haploid cells as compared with diploid cells. To alleviate any concern about the specificity of the relationship between the FA and BLM pathways, the authors could perform a parallel study in a standard diploid FANCC-deficient cell line to knockdown or knockout BLM (or RMI1, RMI2, or TOP3A) and confirm resistance to MMC specifically in the doubly-deficient condition.

The pathologies that result due to mutations in *LMNA* are most likely a result of downstream effects on chromatin structure, gene expression, DNA repair and DNA replication, hence I would agree that LMNA is not primarily a DNA repair gene. This section has been reworded to read: 'To validate the use of HAP1 as a cellular model system in which to identify genetic synthetic viable interactions for genes associated with DNA repair…'; lines 81-84.

With regard to the reviewers concern about the use of haploid cells to study HR, I would like to add that during HR it is the sister chromatid that is used as a template for the damaged strand and hence this DNA repair pathway is restricted to S phase, when such a template is available(*1*). Hence, yeast as a haploid model organism, has proved so useful in studying DNA repair, including HR(*2*). Only in extremely rare and unusual instances is the second allele used as a template. Nevertheless, since mammalian haploid cells are known to frequently diploidize, we have taken advantage of this fact. Except for the genome-wide screens, all FA – BLM interaction experiments were performed using diploid HAP1 cells, as shown in **Figure 1, for Reviewer #1**. If this reviewer feels it necessary, we can add this data to the manuscript.

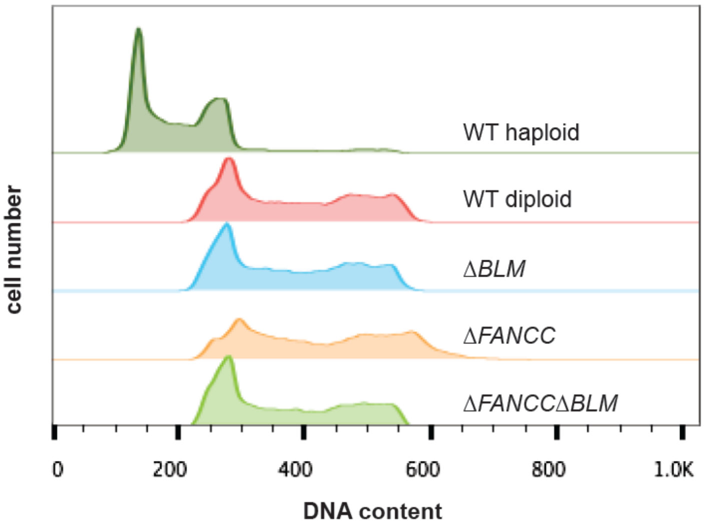

Figure 1, for Reviewer #1

DNA content of wild-type (WT) haploid HAP1 cells as well WT diploid HAP1 cells, along with diploid *ΔBLM, ΔFANCC* and *ΔFANCCΔBLM* cells that were used in all experiments except for the genome-wide screens.

2. (Fig. 1) The evidence showing FANCC targeting and knockout is not very convincing. The Western blot (Fig. 1b) showing FANCC expression is suboptimal due to the low level of signal even in the wild type lane (the same issue exists for knockout of FANCI expression in Fig,. S4g). Either this blot needs to be overexposed or redone so that the control is very prominent and the knockout is completely absent under better exposure conditions. In addition or alternatively, specificity for FANCC deficiency in the viability and colony assays could be strengthened by showing that transfection of wild type FANCC into FANCC cells restores survival after MMC treatment. Also, authors should show a graphical depiction of their colony forming assay results.

We have shown targeting of FANCC and FANCI by both Sanger sequencing of the mutation sites (**Figures S1c and S4f, respectively**) and immunoblotting (**Figures S1d and S4g, respectively; new data**). Moreover, functional validation of these knockout cells has been performed by assessing cellular sensitivities to crosslinking agents (**Figures 1d, S1e-g and 1g**).
As requested, we have now provided graphical depiction of all the colony forming assays that can be found in **Figures S1g, S3g, S5f, S5j, S6c; new data**.

3. While the data presented are intriguing, the endpoint of viability used by the authors is a relatively crude measure of linkages between pathways. A molecular linkage between the FANC and BLM pathways that both impact genome stability would be significantly strengthened by additional experimentation that confirms that deficiency in a BLM complex component rescues the DNA metabolic defect caused by FANC deficiency. For example, does inactivation of BLM complex components help facilitate ICL removal, suppress MMC-induced chromosome aberrations (radial formation) in FANC cells, or suppress double-strand break formation during ICL repair (as suggested in lines 166-168)?
We thank the reviewer for these positive remarks and agree that cell survival is a downstream readout following DNA damage. To understand the molecular mechanisms leading to increased cellular survival, we have now measured generation and kinetics of clearance of DNA damage (using γH2AX; **Figure 2d; new data**), kinetics of apoptosis (using Annexin V; **Figure 2e; new data**) and chromosomal aberrations (via metaphase spreads; **Figure 2f**; **new data**). These data indicate that DNA damage is cleared with faster kinetics in *ΔFANCCΔBLM* cells as compared to *ΔFANCC* hence, leading to reduced apoptosis. However, chromosomal instability is not reduced.
Moreover, in responses to comments raised by Reviewer #3 below, we have ruled out the possibility that other nucleases such as MUS81 can function in the absence of BLM hence leading to enhanced survival after MMC treatment (**Figure S6f; new data**). In addition, we have ruled out the possibility that loss of BLM can rescue defects resulting from ablation of other homologous recombination factors (such as BRCA1) (**Figure S6a-c; new data**). Indeed, we have now supplemented our manuscript with data suggesting that alternative end-joining is at least partially used for this genetic rescue interaction between FA and BLM (**Figures 2g and S6g; new data**).

4. (lines 151-171) The authors should more clearly develop and discuss their ideas about how loss of the BLM complex rescues MMC hypersensitivity caused by deficiency of FANCC or other FA components. In this regard, there is a significant amount of literature linking the BLM and FA pathways, including reports showing physical interaction of BLM with FANC components; very little of this literature is mentioned in the authors' brief treatment here. Also, some of the results suggest near complete suppression of MMC hypersensitivity; it seems highly unlikely that loss of an HR pathway would restore "normalcy" in survival to cells that lack the ICL repair pathway dependent on HR. In regards to specifying the effect of the observed suppression, the authors should also consider the possibility that the BLM dissolvasome complex may be required to relieve topological strain between replication forks converging upon an ICL, an activity potentially necessary to yield a relevant DNA structural intermediate early in crosslink repair. By this reasoning, loss of BLM complex components could thus channel ICL repair into an FA-independent pathway.
We thank the reviewer for his/her suggestion on how loss of the BLM complex could be channeling ICL repair through an FA-independent repair pathway. Based on the data obtained from point 3 above (as well as data from other points raised by Reviewers #2 and #3) we are now able to better clarify the role of BLM in suppressing the cellular sensitivity of FA deficient cells to MMC where we propose, in the current version of the manuscript, that alternative end-joining is used, at least partially, to achieve this rescue interaction.

Minor comments

1. (p. 2, lines 60-74) The authors should clearly define synthetic viability here as the suppression of a genetic defect or phenotype by abrogation of another gene or pathway. Moreover, although the term "synthetic viability" has been used previously, this reviewer feels this is somewhat misleading as the strategy should hopefully suppress the effects of a metabolic defect even under conditions where the affected cell or organism retains viability. Instead, this strategy better reflects identification of "synthetic" suppressors.
We have reworded this section to: 'Synthetic viability is the suppression of a genetic defect or phenotype by abrogation of another gene or pathway. Recently, the concept of utilizing synthetic viability, or synthetic suppression, (either via a gene-gene or gene-drug interaction) as an approach to correct defects in human genetic diseases, including those affecting DNA repair has emerged'; lines 61-62. Moreover, we have used the term 'suppression' throughout the manuscript; lines 39, 40, 43, 51, 63, 76, 161, 188, 212, 239, 569, 585.

2. (Fig. 2c, d) If NQO1 is required for activation of MMC to generate ICLs, clarify why the survival of delta FANCC-deltaNQO1 cells in response to MMC is not identical to wild type cells treated with MMC.
NQO1 is required for MMC to exert its toxicity hence loss-of-function mutations within *NQO1* render cells less sensitive to MMC. This is true for both WT cells and *ΔFANCC* cells i.e. loss of NQO1 renders cells less sensitive (or more resistant) to MMC regardless of genetic background (**Figures S3e-g**). Yet this resistance is partial due to several reasons that we have now clarified in the text:
1. In these experiment we have used a pool of either WT cells or *ΔFANCC* cells that have been exposed to guide RNAs to target NQO1 and most likely these pools of cells contain edited cells that do not carry frameshift mutations within NQO1. See line 116.
2. MMC can be metabolically activated by other enzyme systems including NADPH:cytochrome P450 reductase (*3*) (*4*), cytochrome b5 reductase (*5*), xanthine oxidase/dehydrogenase (*4, 6, 7*). See lines 110-111.
3. Moreover, MMC bioactivation by NQO1 is pH dependent in vitro (*8*), and this pH dependence seems to be due to inactivation of NQO1 by MMC at more neutral pH. Furthermore, evidence suggests that the relationship between NQO1 levels and the toxicity of bioreductive substrates is best described by a threshold relationship, and thus, a simple correlation between activity and toxicity may not exist (*9*).

3. (lines 147-149) The statement "Following exposure to MMC, we observed …" suggests a lack of 'rescue' of WT cells by loss of BLM or RMI1 when these cells are not MMC hypersensitive in the first place and show essentially no change. This statement should be reworded for clarification.
This has been reworded to: 'We observed that loss of RMI1 or BLM could rescue the MMC hypersensitivity of FANCI and FANCD2 deficient cells, but did not enhance MMC resistance in WT cells.' See lines: 158-160.

4. (lines 168-171) This statement appears to be backward. Specifically, depletion of HR factors suppresses deficiencies in the Sgs1-Top3 complex, according to data from the cited reference. Thus, this concept may be less relevant to the scenario described in the current study.
This statement has been removed.

5. References #13 and #23 are incomplete.
These have been removed (point 4 above) or corrected.

Reviewer #2 (Remarks to the Author):

In this interesting paper Loizou et carries out a genome wide synthetic viability screen to look for genes that confer sensitivity to the DNA crosslinking agent MMC to human cells carrying the FA DNA repair activity. The base of of their screen is the haploid CML cell line HAP1 which has been the focus on many recent genetic greens by virtue of the lines haploid genome. Using this approach they pull out an established target - NQO but they also pull out several genes that function in the Blm complex (Blm being mutated in Bloom syndrome and involved in HJ junction resolution). They further validate these targets and also show that this synthetic viability can also be extended to other FA genes particularly those that work downstream of FANCC such as FANCD2 and FANCI. Whilst this is certainly an interesting and potentially important finding it is my view that the analysis and implications of this are much underdeveloped to merit publication in its current form.

We are pleased to have received these encouraging remarks and agree that we did not put forward a molecular link for how loss of BLM was increasing viability of FA cells, in the original version of our manuscript. Having addressed the comments raised by this reviewer in full (and indeed those raised by Reviewers #1 and #3), we now find our manuscript more complete.

1. Te authors finding is not replicated in the chicken B cell line DT40. See EMBO J. 2005 Jan 26; 24(2): 418–427. This is rather intriguing and may argue that the discovery reported here may be specific to HAP1 and possibly due to a haploid genome.
We have confirmed this interaction in diploid HAP1 cells, hence this interaction is not limited to haploid genomes (see **Figure 1, for Reviewer #1** above). However, we cannot rule out the possibility that it is specific to HAP1. Another possibility is that difference occurs due to species variation. This has been discussed; lines 150-152. In support of this, it has been reported that mouse embryonic fibroblasts lacking both FANCB and BLM are less sensitive to MMC than FANCB single mutants(*10*), and since we have shown that the suppression interaction is not limited to FANCC but also extends to FANCI and FANCD2, it is possible that this interaction is not conserved in DT40 cells.

2. Whilst the authors see that whilst Blm deletion suppresses MMC sensitivity in FA mutant HAP1 cells they present no evidence why this suppression occurs. For instance is chromosome breakage mitigated ? is Apoptosis to DNA damage been attenuated ? In addition dose this also suppress sensitivity to other crosslinking agents such as Cis-platin and DEB. In addition the possible physiological source of DNA cx might be the aldehydes - formaldehyde and formaldehyde ?
We have now looked more closely at possible molecular mechanisms of how this suppression occurs. Hence we have measured generation and kinetics of clearance of DNA damage (using γH2AX; **Figure 2d; new data**), kinetics of apoptosis (using Annexin V; **Figure 2e; new data**) and chromosomal aberrations (via metaphase spreads; **Figure 2f; new data**). Please see above 'Specific comments; point 3' raised by Reviewer #1.
We have also tested sensitivity to the crosslinking agents cisplatin and DEB (**Figures 2a-b; new data**), as well as aldehydes (**Figure 2c; new data**) and confirmed the synthetic viable interaction between FANCC and BLM. Also, to strengthen the molecular basis for this rescue, and in response to comments made by Reviewer #3, we have ruled out the possibility that loss of BLM is able to rescue defects resulting from loss of other HR factors (here we selected BRCA1; **Figure S6a-c, new data**). Moreover, we have discarded the possibility that other nucleases such as MUS81 are able to compensate for loss of BLM within our experimental setup by additionally knocking out MUS81 in *ΔFANCCΔBLM* cells (**Figure S6d-f; new data**). Interestingly, however we have obtained data supporting alternative end-joining as a mechanism that alleviates cellular toxicity of *ΔFANCCΔBLM cells* exposed to MMC (**Figures 2g and S6g; new data**).

3. In many cellular and animal systems p53 deletion suppresses the sensitivity of FA repair mutant cells lines to crosslinking agents. It is therefore a surprise that the authors to not pick up p53 or its effectors. This could mean that Hap1 might be defective in p53or indeed in its p53 cell cycle and apoptosis responses. The authors really ought to clarify this, and this is important as knowing the functional p53 status of Hap1 has implications for the many people who use this system. In the end if this cell line has a defect in this and since we know that p53 has such a huge impact on the viability of cells to DNA damage then this fact needs to be established before one draws conclusions about DNA damage impact on cell viability.
We have confirmed a reported mutation in *TP53* carried within HAP1 cells (**Figure S5a; new data**). To test whether p53 is functional in HAP1 cells we generated *ΔTP53* HAP1 cells as well as Δ*FANCC*Δ*TP53* HAP1 cells (**Figures S5b-c; new data**). We tested the response of these cells to MMC in both short-term and long-term dose-response assays to MMC (**Figures S5d-f; new data**). The data from these experiments indicates that Δ*FANCC*Δ*TP53* cells are only slightly more resistant to MMC than *ΔFANCC* cells and hence we would not expect to pick up *TP53* (or components of this pathway) as a hit in our screens. To address if such a small rescue of survival was observed in Δ*FANCC*Δ*TP53* cells because p53 functions are affected in HAP1 cells, we treated HAP1 and A549 cells (as a positive control since this cell line is *TP53* WT) with Nutlin-3a to stabilize p53 and measured the effects on p21 protein levels. While we observed stabilization of p53 and a marked increase in p21 protein levels in A549 cells, this was much reduced in HAP1 cells (**Figure S5g; new data**). This leads us to conclude that p53 activity is much reduced in HAP1 cells, but not entirely absent. In line with this, we did not observe any increased sensitivity of Δ*FANCC*Δ*TP53* cells to Nutin-3a (**Figure S5i-j; new data**).

4. The FANCM gene is part of the FA complex but is also part of the Blm complex. Some functional evidence links the FA pathway to Blm through this interaction. have the authors deleted FANCM if so does this suppress sensitivity ? This is an important point to clarify as the original FANCM patient in fact had a mutation in FANCA and strikingly was only mildly effected - this work here may provide a possible mechanism for this. Indeed see Nat Struct Mol Biol 12 (9), 763-771 here FANCM deletion combined with FANCC suppresses Cx sensitivity.
FANCM was indeed identified as a rescue gene from the genome-wide CRISPR screen. This has now been indicated in **Figures 1b and S4a**, as well as in the text; lines 126-128. Moreover we have generated Δ*FANCC*Δ*FANCM* cells that are also less sensitive to MMC than *ΔFANCC* cells (**Figure 1f, S4d and S4e; new data**).

5. I do not think that blocking Blm is a plausible way to treat FA - Bloom syndrome is much worse illness than FA !In any case point 1 needs to addressed to determine how this suppression works is because DNA damage does not persist or that the response to DNA damage is impacted that also means that point 3 needs to be addressed
We apologize if we appeared to suggest that blocking BLM could be a clinical intervention for the treatment of FA. We have looked over our manuscript more carefully and corrected several sentences that might be misleading as indicated below:

a. Abstract – the following **sentence has been removed**: 'Recently, the concept of synthetic viability has emerged to ameliorate pathologies that occur in rare diseases'. The following **sentence as been modified** as indicated: 'hence demonstrating that systematic genome-wide screening approaches can be used to reveal genetic viable interactions for ~~disease-associated~~ DNA repair defects'.
b. Main text – the following **sentence has been removed**: 'For many rare diseases associated with defects in DNA repair, including FA, no specific treatment options exist'. The following **section has been removed**: 'Importantly, synthetic viability-inducing agents offer potential for new treatment modalities for rare genetic diseases that may be more safe and effective than current strategies such as bone marrow transplantations, protein replacement therapies or gene therapies. Unfortunately, so far there has been slow

progress in identifying synthetic viable interactions due to the lack of unbiased identification approaches'.

Reviewer #3 (Remarks to the Author):

The manuscript by Moder et al. reports the use of CRSIPR and insertional mutagenesis screens in haploid cell lines to identify synthetic viable interactions with deletion of FANCC, a protein of the Fanconi anaemia pathway. The screening protocol described in the paper is robust and certainly will be of interest to the Fanconi and DNA damage response field. The authors first establish the suitability of HAP1 cells for synthetic viable interactions with DNA repair factors by recapitulating previously reported remodelin (NAT10 inhibitor) reversal of ageing phenotypes in cells lacking lamin A. Then, they identify a novel and exciting synthetic viable interaction between FANCC and BLM in response to MMC treatment. Two separate approaches are used for screening: CRISPR/Cas9 genome-wide library and insertional mutagenesis. Both led to identification of NQO1, a reductase previously shown to rescue MMC-induced lethality in Fanconi cells. Furthermore, deletion of BLM interacting partners RMI1, RMI2 or TOP3A can reverse MMC toxicity in FANCA-deleted cells. Thus, antagonism between the Fanconi pathway and BLM complex in the repair of MMC-induced damage is clearly demonstrated and will help characterise mechanisms of drug resistance informative to the clinic.

We are pleased that the reviewer found our approaches robust and the outcome of our genome-wide screens to be interesting.

The problem however, is that the paper lacks mechanistic insight, apart from two speculative scenarios described in Discussion. Moreover, as the paper focuses on a genetic screen, the top hits obtained from the screen should be reported in full, as it is customary for this type of papers. Therefore, I am not sure that Nature Communication is the most suitable journal for publication of this work.

We have put quite some effort into understanding the molecular mechanism of the described rescue interaction between FA and BLM, in response to the comments made by Reviewers #1 and #2. Moreover, we have address all the points raised by Reviewer #3. In summary, in the current version of the manuscript we have included generation and clearance of DNA damage as well as kinetics of apoptosis, we have investigated effects on chromosomal aberrations, we have tested other DNA damaging agents including aldehydes (that are considered to represent the endogenous source of crosslinks in FA cells) and we have unraveled the contribution of p53 to our findings.
Moreover, as requested by this reviewer, we have ruled out the hypothesis that other nucleases (we focused on MUS81, as suggested) can compensate for lack of BLM in FA deficient cells and we have also ruled out the hypothesis that BLM can rescue depletion of other HR factors (we selected BRCA1). Also we have identified alternative end-joining to be, at least partially, required for the observed rescue effect. Based on these data (displayed in 2 main figures as is required for a brief communication; as well as 6 supplemental figures) we have now better developed the working model of this rescue as expanded on in the manuscript.
Also, in the current version of the manuscript, in our opinion the focus of the manuscript is not on the data resulting from the genetic screens but on the validation and characterization of the BLM complex in rescuing FA-dependent cellular sensitivities to DNA crosslinking agents.

Specific comments:

Introduction:
p.2 Line 61: It should be further explained that cancers in FA patients cannot be treated with conventional chemotherapy, such as platinum drugs due to their increased sensitivity.

This has been clarified to read: 'Moreover, FA patients that develop cancer cannot be treated with standard chemotherapy, including crosslinking agents, as they are hypersensitive to such compounds', lines 57-59.

Results:

p.4 line 121: Cells showed reduced toxicity, but not resistance in Figure 2d

This has been rephrased to: 'Both WT and *ΔFANCC* cells targeted for NQO1 ('WT + *NQO1* gRNA' and '*ΔFANCC* + *NQO1* gRNA', respectively) displayed reduced toxicity to MMC in both a short-term dose response assay (**Figure S3e**) and a long-term colony formation assay (**Figure S3f**)', lines 117-120.

p. 4 line 122: It should be explained in more detail that NQO1 does not represent a true "synthetic viable" interaction with the FA pathway. Pathologies seen in FA patients mostly arise from their inability to resolve endogenously formed ICLs or stalled replication forks. Inactivation of NQO1 however is only able to rescue FA-deficient cells, which are treated with the exogenous agent MMC, therefore this interaction may not be physiologically relevant.

We have clarified the fact that within our experimental system NQO1 functions specifically to induce toxicity of MMC. It is for this reason that when loss-of-function mutations are generated within *NQO1*, cells are more resistant to MMC (be it WT cells or *ΔFANCC* cells). This is apparent from the dose responses in **Figures S3e-g**. The identification of NQO1 under our screening conditions were cells are treated with MMC is certainly not physiologically relevant, but acts as a positive control to indicate that our screens work (we can identify factors that reduce toxicity to MMC) and that we can identify known candidates that function within the system we are utilizing. To not distract from our main finding (the BLM complex), that is specific to FA mutant cells, all data on NQO1 is now displayed entirely as supplementary.

p. 4 line 123: The complete list of synthetic viable interactions, which were identified in the two screens described, should be given.

Since the focus of this manuscript is not on the data resulting from the genetic screens but rather on the validation and characterization of the BLM complex in rescuing FA-associated defects, we do not think that providing a full list of hits from both screens, without any further analysis of these hits, will improve the manuscript. However, in order to address these concerns, we are providing this list here as an excel sheet. [Editorial Note: The authors have provided this file directly to the reviewers and asked it not be made public.]

p. 4. line 144: BLM or RMI1 could rescue "cells lacking" FANCI and FANCD2

We thank the reviewer for noticing this. This has been corrected and now reads: 'Since FANCC is part of the FA core complex, we next investigated whether loss of BLM or RMI1 could rescue cells lacking FANCI and FANCD2 that make up the FA I-D2 complex and function downstream of the FA core complex'; lines 154-156.

Discussion:

The authors hypothesize that the absence of the BLM complex leads to preferential resolution of ICLs by structure specific nucleases (MUS81 or GEN1), which promotes survival of FA-pathway deficient cells. The authors could test this by depleting MUS81 or GEN1 in cells lacking FANCA and BLM, and analyse if depletion of these factors re-sensitizes cells to MMC.

We have tested this by targeting MUS81 using CRISPR-Cas9 in a *ΔFANCCΔBLM* background (**Figure S6d-f; new data**). Indeed we did not observe a re-sensitization of these cells to MMC, indicating that MUS81 is not promoting cell survival in *ΔFANCCΔBLM* cells. However, we have included data in the manuscript that highlights the role of alternative end-joining in at least partially leading to the resistance in *ΔFANCCΔBLM* cells (**Figures 2g and S6g**; **new data**).

Furthermore, the authors hypothesize that BLM contributes to the formation of double strand breaks, which requires the HR for their repair. It is therefore conceivable that BLM inactivation also

rescues cells lacking HR factors, such as BRCA2. This could be tested in clonogenic survival assays.
Since many HR factors are essential we have taken an shRNA approach where we have knocked-down BRCA1. We have not observed that knock-down of BRCA1 in cells lacking BLM leads to less sensitive to MMC, hence potentially loss of BLM does not function to alleviate toxicity to MMC as a general mechanism for HR factors (**Figure S6a-c; new data**).

Furthermore, it would be interesting to test whether BLM inhibition using chemical inhibitors (eg. ML261) could recapitulate the synthetic viable interaction between BLM and FANCC.
We have tested the BLM inhibitor ML261, published by the Hickson lab(*11*). We have observed a reduction in cell viability upon using this inhibitor on *ΔBLM* cells (**Figure 2a, for Reviewer #3 below**). This could be because it is not specific for BLM (it also targets WRN) (*11, 12*) or it could be functioning to inhibit BLM on DNA and hence preventing the binding of other nucleases such as WRN compensating for the lack of BLM activity (similar to what is now about the ATM inhibition verses ATM loss(*13*). These issues would need to be addressed before drawing conclusions on the use of this inhibitor.
Nevertheless, we have tested whether this inhibitor can rescue *ΔFANCC* cells but we observed an increased sensitivity that could be explained by the reasons above. We provide this data in the following figure (**Figure 2b, for Reviewer #3 below**) but do not think it should become part of the manuscript.

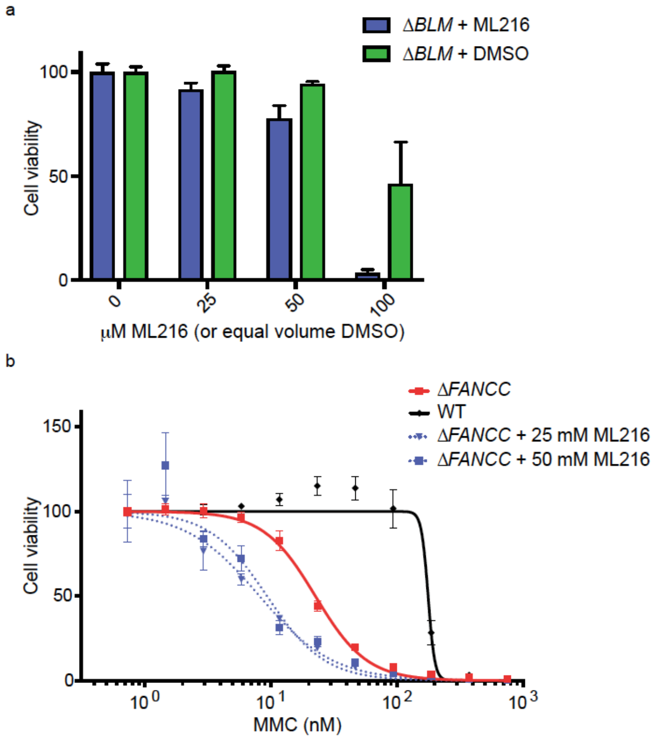

Figure 2, for Reviewer #3
a. *ΔBLM* cells were incubated with the BLM inhibitor ML216 at the indicated concentrations (or an equivalent volume of DMSO, as a vehicle) for 4 days following measurement of survival by CellTiter-Glo. b. The indicated cell lines were incubated with the BLM inhibitor ML216 for 24 hours and then exposed to MMC for 4 days. Cell survival was measured by CellTiter-Glo.

Should you require any further information from me please do not hesitate to contact me.
I look forward to hearing from you.

Best regards,
Joanna LOIZOU

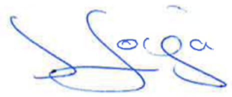

References:

1. J. Matos, S. C. West, Holliday junction resolution: regulation in space and time. *DNA repair* **19**, 176 (Jul, 2014).
2. L. S. Symington, R. Rothstein, M. Lisby, Mechanisms and regulation of mitotic recombination in Saccharomyces cerevisiae. *Genetics* **198**, 795 (Nov, 2014).
3. S. R. Keyes *et al.*, Role of NADPH:cytochrome c reductase and DT-diaphorase in the biotransformation of mitomycin C1. *Cancer research* **44**, 5638 (Dec, 1984).
4. S. S. Pan, P. A. Andrews, C. J. Glover, N. R. Bachur, Reductive activation of mitomycin C and mitomycin C metabolites catalyzed by NADPH-cytochrome P-450 reductase and xanthine oxidase. *The Journal of biological chemistry* **259**, 959 (Jan 25, 1984).
5. W. F. Hodnick, A. C. Sartorelli, Reductive activation of mitomycin C by NADH:cytochrome b5 reductase. *Cancer research* **53**, 4907 (Oct 15, 1993).
6. D. L. Gustafson, C. A. Pritsos, Enhancement of xanthine dehydrogenase mediated mitomycin C metabolism by dicumarol. *Cancer research* **52**, 6936 (Dec 15, 1992).
7. D. L. Gustafson, C. A. Pritsos, Bioactivation of mitomycin C by xanthine dehydrogenase from EMT6 mouse mammary carcinoma tumors. *Journal of the National Cancer Institute* **84**, 1180 (Aug 05, 1992).
8. D. Siegel, N. W. Gibson, P. C. Preusch, D. Ross, Metabolism of mitomycin C by DT-diaphorase: role in mitomycin C-induced DNA damage and cytotoxicity in human colon carcinoma cells. *Cancer research* **50**, 7483 (Dec 01, 1990).
9. S. L. Winski, R. H. Hargreaves, J. Butler, D. Ross, A new screening system for NAD(P)H:quinone oxidoreductase (NQO1)-directed antitumor quinones: identification of a new aziridinylbenzoquinone, RH1, as a NQO1-directed antitumor agent. *Clinical cancer research : an official journal of the American Association for Cancer Research* **4**, 3083 (Dec, 1998).
10. T. M. Kim *et al.*, RECQL5 and BLM exhibit divergent functions in cells defective for the Fanconi anemia pathway. *Nucleic acids research* **43**, 893 (Jan, 2015).
11. G. H. Nguyen *et al.*, A small molecule inhibitor of the BLM helicase modulates chromosome stability in human cells. *Chemistry & biology* **20**, 55 (Jan 24, 2013).
12. T. Banerjee, M. Aggarwal, R. M. Brosh, Jr., A new development in DNA repair modulation: discovery of a BLM helicase inhibitor. *Cell cycle* **12**, 713 (Mar 01, 2013).
13. J. A. Daniel *et al.*, Loss of ATM kinase activity leads to embryonic lethality in mice. *The Journal of cell biology* **198**, 295 (Aug 06, 2012).

Reviewers' Comments:

Reviewer #1:
Remarks to the Author:
The manuscript "Parallel genome-wide screens identify a synthetic viable interaction between the BLM helicase complex and Fanconi anemia" by Moder et al. uses CRISPR and insertional mutagenesis technologies to show that loss of function of BLM (or associated factors RMI1, RMI2, and TOP3A) rescues the sensitivity of FA-deficient cells to induction of interstrand crosslinks by MMC (or other agents). The authors' data clearly shows that deletion of BLM complex components rescues the sensitivity of FA-deficient cells to interstrand crosslinking agents. However, the molecular explanation for this remains completely unclear, despite the inclusion of new experiments in this revision. To the authors' credit, new experiments showing the involvement of FANCM, the effect of p53 status, and the possible utilization of alternative end joining in ICL repair in the absence of the FA pathway and BLM add more depth to the results. However, other experiments designed to address the molecular aspects of this relationship are unconvincing (see specific comments #2 and 3), and thus do not bring any clarity to this issue. In addition, the authors' fail to put their results in any context in relation to published knowledge about ICL repair, the FA pathway, and BLM function.

Specific comments:

1. In their response to reviewer 1, point 1, the authors claim that endpoints in certain experiments were achieved in diploid cells instead of haploid cells used to generate the gene knockouts, although this is not expressly stated at any relevant points in the manuscript. For clarity, the use of diploid cells for specific experiments needs to be clearly stated in the main text, while their derivation from haploid cells should appear at least in the Methods if not in the main text.

2. (p. 6, Fig. 2d-f) There are several concerns about the mechanistic data included in this revision and the authors' interpretation of this data. Although the depiction of data using 2-4 asterisks might imply significant differences (note that the degree of significance is also undefined for both panels d and f in the legend), no error bars are shown for H2AX data and the small magnitude of the differences shown for this endpoint calls into question the authors' conclusion that "clearance of DNA damage at a later time was accelerated in deltaFANCCdeltaBLM cells compared to deltaFANCC cells." Similar concerns also apply to the apoptosis and chromosomal aberrations data in Fig. 2e and f. Perhaps most troubling is the authors statement that "shortly after ICL induction, the number of chromosomal breaks and gaps in deltaRANCCdeltaBLM cells was increased compared to deltaFANCC" when the statistical analysis shows no significant difference between these conditions. Additional data or detail needs to be included for these results and their interpretation to be validated and to clarify the molecular relationship between the FA pathway and BLM.

3. (p. 6, Fig. S6a-f) Experiments using cells knocked down for BRCA1 are unreliable, based on the expression data in Fig. S6a that show the deltaBLMshBRCA1 cells retain 25-30% BRCA1 expression. This substantial level of BRCA1 expression in the BLM-deficient background may be enough to render the cells fully or mostly functional for BRCA1. In that case, comparisons between deltaBLM and deltaBLMshBRCA1 shown in Fig. S6b-c are untenable. Somewhat of a similar concern exists with the set of experiments involving deletion of MUS81. Specifically, the Western blot (Fig. S6e) of the deltaFANCCdeltaBLMdeltaMUS81pool condition shows some retention of MUS81 expression, thus rendering comparisons of this condition with the deltaFANCCdeltaBLM condition (Fig. S6f) somewhat untenable. Although it is unclear from the details presented, presumably some cells in this "pool" sample have retained wild type MUS81.

4. There is essentially no discussion of how the cumulative results shown fit into what is currently known about ICL repair, the FA pathway, and BLM function. There should be at least one substantial paragraph devoted to putting these findings into some context.

Minor comments

1. (p. 4) As written, the statement “these proteins functions in the resolution of DNA structures that arise during the process of homologous recombination repair” appears to refer to not only the BLM complex but also FA proteins and is thus misleading, as many FA proteins participate in signaling and protein modification reactions that precede crosslink repair steps but do not themselves directly alter DNA structures. Thus, this statement should be moved or modified to specify that resolution of DNA structures is, in this context, relevant only to the BLM complex.

2. (p. 4, lines 126-127) The statement “The BLM complex is bridged to the FA complex via FANCM” needs to be cited.

3. (p. 4, Fig. S4b) The authors state that “all six gRNAs for BLM and RMI1 were enriched in the CRISPR screen,” however the RMI1 panel of Fig. S4b seems to only show data for 5 gRNAs.

4. (Fig. S4d) The Exon 2 FANCM mutation is depicted erroneously as additional “K” within the sequence.

5. (p. 5, line 145) Use “one” instead of “either” which is grammatically incorrect when there are more than 2 alternatives.

6. (p. 7, lines 227-230) Use of the term “ICL intermediates” in this context is inappropriate, since functionality of the FA core and FANCD2-FANCI complexes are necessary to even generate standard ICL repair intermediates. The text here should be revised to simply indicate that combined FA and BLM deficiency may channel removal of intact ICLs into other unrelated pathways that may involve alternative end joining factors such as PARP.

Reviewer #2:
Remarks to the Author:
Overall the authors have addressed my concerns but must take them up on rather sloppy referencing lines 145-152. Firstly the suppression of FANCM deletion on the sensitivity on FANCC deficient cells has been reported before and this should be both discussed and referenced (Nature Structural & Molecular Biology 12, 763 - 771 (2005) figure 3 and discussion). Secondly,in lines 183 -184 the statement that endogenous crosslinks might be caused by acetaldehyde is not referenced and this should be.

Other than this the work is now acceptable for publication

Reviewer #3:
Remarks to the Author:
The authors had made quite an effort to address the reviewers comments and have added a lot of new data. Their conclusions are now better supported by experimental evidence. However, it remains questionable whether introducing another DNA repair defect (Blm deficiency) is a good therapeutic strategy for Fanconi anemia patients. Thus, I believe that their findings are not therapeutically relevant, so this aspect could be toned down further.

Reviewer #1 (Remarks to the Author):

The manuscript "Parallel genome-wide screens identify a synthetic viable interaction between the BLM helicase complex and Fanconi anemia" by Moder et al. uses CRISPR and insertional mutagenesis technologies to show that loss of function of BLM (or associated factors RMI1, RMI2, and TOP3A) rescues the sensitivity of FA-deficient cells to induction of interstrand crosslinks by MMC (or other agents). The authors' data clearly shows that deletion of BLM complex components rescues the sensitivity of FA-deficient cells to interstrand crosslinking agents. However, the molecular explanation for this remains completely unclear, despite the inclusion of new experiments in this revision. To the authors' credit, new experiments showing the involvement of FANCM, the effect of p53 status, and the possible utilization of alternative end joining in ICL repair in the absence of the FA pathway and BLM add more depth to the results. However, other experiments designed to address the molecular aspects of this relationship are unconvincing (see specific comments #2 and 3), and thus do not bring any clarity to this issue. In addition, the authors' fail to put their results in any context in relation to published knowledge about ICL repair, the FA pathway, and BLM function.

Specific comments:

1. In their response to reviewer 1, point 1, the authors claim that endpoints in certain experiments were achieved in diploid cells instead of haploid cells used to generate the gene knockouts, although this is not expressly stated at any relevant points in the manuscript. For clarity, the use of

diploid cells for specific experiments needs to be clearly stated in the main text, while their derivation from haploid cells should appear at least in the Methods if not in the main text.

We thank the reviewer for pointing out this lack of clarity. We have now indicated in the main text in which experiments haploid or diploid cells were used (Lines 71-76). Moreover, we have indicated how diploid cells were derived from haploid cells, in the methods section of the manuscript. Lines 275-278.

2. (p. 6, Fig. 2d-f) There are several concerns about the mechanistic data included in this revision and the authors' interpretation of this data. Although the depiction of data using 2-4 asterisks might imply significant differences (note that the degree of significance is also undefined for both panels d and f in the legend), no error bars are shown for H2AX data and the small magnitude of the differences shown for this endpoint calls into question the authors' conclusion that "clearance of DNA damage at a later time was accelerated in deltaFANCCdeltaBLM cells compared to deltaFANCC cells." Similar concerns also apply to the apoptosis and chromosomal aberrations data in Fig. 2e and f. Perhaps most troubling is the authors statement that "shortly after ICL induction, the number of chromosomal breaks and gaps in deltaFANCCdeltaBLM cells was increased compared to deltaFANCC" when the statistical analysis shows no significant difference between these conditions.
Additional data or detail needs to be included for these results and their interpretation to be validated and to clarify the molecular relationship between the FA pathway and BLM.

We apologize for the lack of clarity regarding Figure 2. We have made the following changes to Figure 2 and the associated text:

1. In the legend of Figure 2 we have now indicated the significance represented by 1-4 asterisks.
2. Following reanalysis of the data in triplicates, error bars and statistical significance have been added for the γH2AX immunofluorescence. Because there is a statistically significant difference in the levels of γH2AX in Δ*FANCC*Δ*BLM* cells compared to Δ*FANCC* cells at 48 hours and 72 hours after MMC treatment, our conclusions remain unchanged.
3. The apoptosis experiment was repeated using biological triplicates and error bars, indicating the S.E.M. were added to the graph. Our conclusions remain unchanged.
4. Error bars have been added for the chromosomal aberrations (statistical significance had been added in the previous version of our manuscript). We were leaning on the side of caution by pointing out that if anything, chromosomal aberrations were increased in Δ*FANCC*Δ*BLM* cells compared to Δ*FANCC* cells. Yet as the reviewer points out (and as we indicated in the previous version and current version of our manuscript) this increase is not statistically significant. To avoid confusion, we have corrected this part of the manuscript to read: 'shortly after ICL induction (24h after MMC treatment), the number of chromosomal breaks and gaps in Δ*FANCC*Δ*BLM* cells were not significantly altered, compared to Δ*FANCC*'. Line 197-199.

3. (p. 6, Fig. S6a-f) Experiments using cells knocked down for BRCA1 are unreliable, based on the expression data in Fig. S6a that show the deltaBLMshBRCA1 cells retain 25-30% BRCA1 expression. This substantial level of BRCA1 expression in the BLM-deficient background may be enough to render the cells fully or mostly functional for BRCA1. In that case, comparisons between deltaBLM and deltaBLMshBRCA1 shown in Fig. S6b-c are untenable. Somewhat of a similar concern exists with the set of experiments involving deletion of MUS81. Specifically, the Western blot (Fig. S6e) of the deltaFANCCdeltaBLMdeltaMUS81pool condition shows some retention of MUS81 expression, thus rendering comparisons of this condition with the

deltaFANCCdeltaBLM condition (Fig. S6f) somewhat untenable. Although it is unclear from the details presented, presumably some cells in this "pool" sample have retained wild type MUS81.

The aim of the experiments in Figures S6a-c was to address whether loss of BLM can alleviate cellular sensitivities of other homologous recombination proteins to MMC. The data shows that wildtype cells retaining 10% expression levels of BRCA1 are mildly sensitive to MMC. *ΔBLM* cells retaining 30% expression levels of BRCA1 are equally sensitive to MMC. Hence we can make the interpretation that loss of BLM does not alleviate cellular sensitivities of BRCA1 depleted cells to MMC. If the knockdown efficiency were inverted (i.e. 10% BRCA1 expression remaining in *ΔBLM* cells) then I would agree that we cannot draw conclusions from this experiment but since we actually have more BRCA1 expression in *ΔBLM* cells and yet the cells are still not more resistant to MMC we can rule out the possibility that these cells are mostly or fully functional for BRCA1.

The aim of the experiments in current **Figures S6d-e (new data)** was to address whether the loss of other nucleases can re-sensitize *ΔFANCCΔBLM* cells to MMC. To satisfy the concerns of the reviewer, we have taken an alternative approach where we have knocked down MUS81 using two independent shRNAs. Both shRNAs reduced the protein levels of MUS81 efficiently and to a similar level and both shRNAs targeting MUS81 have no effect on the sensitivity of *ΔFANCCΔBLM* cells to MMC. Hence, we can conclude that depletion of MUS81 does not re-sensitize *ΔFANCCΔBLM* cells to MMC.

4. There is essentially no discussion of how the cumulative results shown fit into what is currently known about ICL repair, the FA pathway, and BLM function. There should be at least one substantial paragraph devoted to putting these findings into some context.

Two paragraphs have now been added that put our findings in context to the current state of art. Lines 241-260.

Minor comments

1. (p. 4) As written, the statement "these proteins functions in the resolution of DNA structures that arise during the process of homologous recombination repair" appears to refer to not only the BLM complex but also FA proteins and is thus misleading, as many FA proteins participate in signaling and protein modification reactions that precede crosslink repair steps but do not themselves directly alter DNA structures. Thus, this statement should be moved or modified to specify that resolution of DNA structures is, in this context, relevant only to the BLM complex.

This has been corrected to read: 'The BLM complex functions in the resolution of DNA structures that arise during the process of homologous recombination (HR) repair'. Lines 127-129.

2. (p. 4, lines 126-127) The statement "The BLM complex is bridged to the FA complex via FANCM" needs to be cited.

This reference has now been cited. Line 125-126.

3. (p. 4, Fig. S4b) The authors state that "all six gRNAs for BLM and RMI1 were enriched in the CRISPR screen," however the RMI1 panel of Fig. S4b seems to only show data for 5 gRNAs.

We thank the reviewer for pointing this out. In the original graph, the data points for two gRNAs were overlapping and hence it appeared as though only five gRNAs were depicted. Hence, we have changed the way we have displayed this data to avoid the overlap of data points. For consistency, we have displayed the depiction of gRNA enrichment for NQO1 in Figure S3a in the same way.

4. (Fig. S4d) The Exon 2 FANCM mutation is depicted erroneously as additional "K" within the sequence.

We have clarified this to indicate the *ΔFANCM* clone carries a heterozygous insertion, resulting in a frameshift mutation in both alleles.

5. (p. 5, line 145) Use "one" instead of "either" which is grammatically incorrect when there are more than 2 alternatives.

This has been changed to 'one'. Line 145.

6. (p. 7, lines 227-230) Use of the term "ICL intermediates" in this context is inappropriate, since functionality of the FA core and FANCD2-FANCI complexes are necessary to even generate standard ICL repair intermediates. The text here should be revised to simply indicate that combined FA and BLM deficiency may channel removal of intact ICLs into other unrelated pathways that may involve alternative end joining factors such as PARP.

This has been changed to: 'allow for the use of this pathway in the removal of ICL in FA-deficient cells'. Line 233-234.

Reviewer #2 (Remarks to the Author):

Overall the authors have addressed my concerns but must take them up on rather sloppy referencing lines 145-152. Firstly the suppression of FANCM deletion on the sensitivity on FANCC deficient cells has been reported before and this should be both discussed and referenced (Nature Structural & Molecular Biology 12, 763 - 771 (2005) figure 3 and discussion). Secondly,in lines 183 -184 the statement that endogenous crosslinks might be caused by acetaldehyde is not referenced and this should be.

We thank the reviewer for pointing out the omission of this reference. The publication describing the suppression of FANCC-dependent sensitivity by loss of FANCM (also known as Protein Hef ortholog) in DT40 cells has been cited and discussed. See lines 147-149.

Also we apologize for omitting the references indicating that aldehydes are considered to represent endogenous sources of crosslinking damage. Three references have been added from the Patel lab. See lines 186-188.

Other than this the work is now acceptable for publication

We are pleased that otherwise this reviewer now finds this work suitable for publication and indeed the constructive comments raised by this reviewer have strengthened our manuscript.

Reviewer #3 (Remarks to the Author):

The authors had made quite an effort to address the reviewers comments and have added a lot of new data. Their conclusions are now better supported by experimental evidence. However, it remains questionable whether introducing another DNA repair defect (Blm deficiency) is a good therapeutic strategy for Fanconi anemia patients. Thus, I believe that their findings are not therapeutically relevant, so this aspect could be toned down further.

We thank the reviewer for these positive comments with regard to the substantiation of our conclusions as a result of the new data added to the revised version of our manuscript.

We do agree, that loss of BLM in FA-deficient cells would not be a viable therapeutic option and we did not intend to make this claim. Therefore, we have made the following alterations to the manuscript to avoid confusion:

1. In the introduction, the following section on synthetic viable interactions (and associated references) to correct defects in human genetic diseases has been removed: ‘Recently, the concept of utilizing synthetic viability, or genetic suppression, (either *via* a gene-gene or gene-drug interaction) has emerged as an approach to correct defects in human genetic diseases, including those affecting DNA repair[4-7]. Moreover, there is considerable interest in synthetic-viable relationships because they can provide key insights into mechanisms of cell biology and disease’.
2. Line 262-264: ‘In conclusion, through the use of parallel genome-wide screens, we have shown that synthetic viable (genetic suppression) interactions for disease-associated mutations can be systematically identified in human cells’ has been changed to ‘In conclusion, through the use of parallel genome-wide screens, we have shown that synthetic viable (genetic suppression) interactions for Fanconi anemia can be systematically identified in human cells’.
3. The following concluding sentence has been deleted: ‘This approach could be extended to additional disease-relevant mutations and might reveal novel approaches for therapeutic interventions’.

Should you require any further information from me please do not hesitate to contact me.
I look forward to hearing from you.

Best regards,
Joanna LOIZOU

Reviewers' Comments:

Reviewer #1:
Remarks to the Author:
The manuscript by Moder et al. is significantly improved after the last round of revisions. However, I would suggest the modest change to the appearance of Fig. 2e recommended below.

Minor comments:

1. In the authors' new depiction of Figure 2e that includes error bars within the stacked bar graphs, it might be visually preferable to widen the bar sizes and include the percentages and errors as numerical values (% plus/minus S.E) within the bars themselves, instead of placing error bars on top of each segment.

Please find following the comments raised by the Reviewer in *italics* with our reply in regular text.

Reviewer #1 (Remarks to the Author):

The manuscript by Moder et al. is significantly improved after the last round of revisions. However, I would suggest the modest change to the appearance of Fig. 2e recommended below.

Minor comments:

1. In the authors' new depiction of Figure 2e that includes error bars within the stacked bar graphs, it might be visually preferable to widen the bar sizes and include the percentages and errors as numerical values (% plus/minus S.E) within the bars themselves, instead of placing error bars on top of each segment.

We have now widened the bar sizes. However, since some of the bars, especially the ones representing cells in late apoptosis are vertically narrow, it is not possible to appropriately incorporate the percentage values ± S.E.M. into the bars. A possible graph with incorporated values is shown:

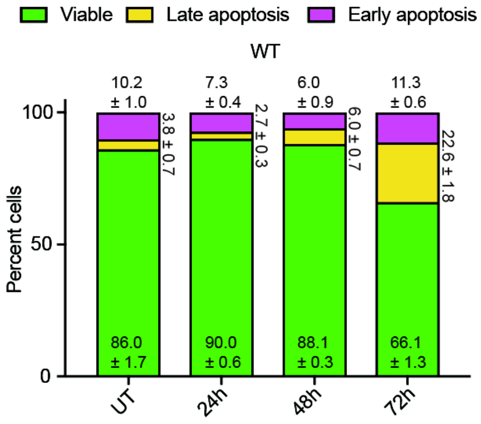

CeMM Research Center for Molecular Medicine of the Austrian Academy of Sciences
Lazarettgasse 14, AKH BT 25.3, 1090 Vienna, Austria, Phone +43-1/40160-70011, office@cemm.oeaw.ac.at, www.cemm.at

We consider a depiction including error bars as more intuitive (as in Figure 2e in the manuscript).

Instead of incorporating the values into the graph, we therefore included a Supplementary Table (Supplementary Table 2) that states the requested values numerically.

		**Viable**		**Late apoptosis**		**Early apoptosis**	
	**Time point**	**Mean**	**S.E.M.**	**Mean**	**S.E.M.**	**Mean**	**S.E.M.**
ΔWT	**UT**	86.0	1.7	3.8	0.7	10.2	1.0
	**24h**	90.0	0.6	2.7	0.3	7.3	0.4
	**48h**	88.1	0.3	6.0	0.7	6.0	0.9
	**72h**	66.1	1.3	22.6	1.8	11.3	0.6
ΔBLM	**UT**	81.4	0.6	6.0	1.0	12.6	1.4
	**24h**	87.7	0.8	4.3	0.4	8.0	0.5
	**48h**	78.7	1.9	12.8	1.6	8.5	0.3
	**72h**	62.1	2.4	26.5	1.7	11.4	1.1
ΔFANCC	**UT**	71.3	1.7	3.5	0.1	25.3	1.8
	**24h**	68.9	0.5	4.6	0.7	26.5	0.6
	**48h**	37.4	1.3	33.1	0.6	29.5	1.9
	**72h**	15.4	1.1	63.6	1.7	21.1	0.7
ΔFANCCΔBLM	**UT**	80.0	1.9	4.8	0.5	15.2	2.0
	**24h**	81.0	0.9	4.8	0.7	14.2	1.5
	**48h**	74.2	1.2	11.6	0.8	14.2	1.6
	**72h**	41.1	1.8	37.4	3.2	21.5	1.9

Supplementary Table 2. Table numerically showing the percentage of cells that are viable, in late or early apoptosis as graphically depicted in Fig. 2e.
